# Supplementary material for: Infant Care Practices, Caregiver Awareness of Safe Sleep Advice and Barriers to Implementation: A Scoping Review
Source: Int J Environ Res Public Health. 2022 Jun 23;19(13):7712. doi: 10.3390/ijerph19137712 (PMC9265757; doi:10.3390/ijerph19137712)
Supplement: Supplementary file 1 [file ijerph-19-07712-s001.zip › ijerph-1725712-supplementary.pdf]

Supplementary Material

Table S1: Overview of scoping review articles

Citation	Country	Study period	Study aim	Sample population	Sample size	Design/method	Key findings
Abdulrazzaq et al (2008)[1]	UAE	July 2005-May 2006	To monitor infant care practice associated with SIDS and establish the incidence of SIDS in the UAE.	families with new babies	n=996 (n=716 completing both surveys)	Quantitative survey<br><br>Via two surveys - one via individual face-to-face interview completed during first 7 days after delivery and another completed after 12 weeks via telephone interview.	18.9% infants placed prone<br>▪49.3% preferred supine position to other positions when putting baby to bed<br>▪98% preferred their infant sleep in same room as parents<br>▪40% occasionally shared their bed with their infant<br>▪23.9% always shared their bed with their infant<br>▪24% never shared their bed with their infant<br>▪70% of either parent did not smoke (18.7% were heavy smokers, 11.3% light smokers)<br>▪83.2% swaddled their baby<br>▪>80% used bedding duvets for their infant both in summer and winter<br>▪66% of UAE mothers used soft pillows
Aitken et al (2016)[2]	US	Not reported - data collected over a 9-month period	To determine any association between grandmothers' beliefs/opinions and family demographic characteristics with following key AAP-recommended sleep guidelines when bedding the infant at the grandmother's house and at the mother's house	Current grandmothers aged 30-70 who were regular [at least once per week] caregivers of an infant grandchild <6months	n=260	Prospective cross-sectional survey: online and paper questionnaires	▪45% place infant supine on appropriate sleep surface at grandmother's house<br>▪58% place infant supine on appropriate sleep surface at mother's house<br>▪34% sleep infants on adult's beds or other surfaces when at grandmother's house compared to 9% when infant cared for at mother's house<br>▪66% sleep in crib, portable cot, playpen, play yard when cared for at grandmother's house compared to 90% when at mother's house<br>▪4% are placed on a couch/chair when at grandmother's house compared to 1% at mother's house<br>▪60% infants placed on back to sleep when at grandmother's house, 62% when at mother's house<br>* grandmothers who believe babies choke on their back are significantly less likely to follow guidelines (OR 0.25, CI 0.13–0.48) at the mother's house.
Ajao et al (2011)[3]	US; Washington DC, and Maryland.	July 2006-Dec 2008	To examine factors influencing decisions by black parents regarding use of soft bedding and sleep surfaces for their infants.	black parents with infants 0 to 6 months of age	n= 83 mothers 13 focus groups; 10 individual interviews	Qualitative study Cross-sectional sample; 2 different qualitative interview formats: focus groups & individual, in-depth, semi-structured interviews,	* primary reason for using soft surfaces was infant comfort<br>*primary reasons for using soft bedding (including bumper pads) were comfort, safety, and aesthetics.<br>*bedding to soften sleep surfaces, bedding was used to prevent infant rollover and falls,<br>* mothers often used pillows to make the sleep surface softer or more comfortable (Table 5). The other primary reason for pillow use was infant safety -used pillows with infants sleeping on beds or sofas to create a barricade around the infant so that he or she would not fall<br>*many mothers had the perception that firm means taut and that the surface would still be firm if a pillow or blanket was placed between the mattress and the sheet, as long as the sheet was tucked tautly around the pillow or blanket.
Alahmadi et al (2020)[4]	Jeddah, Saudi Arabia	not reported	To assess caregivers' implementation of safe sleep practices and if they received any safe sleep education through health care worker	mothers who had children below the age of one-year-old visiting the outpatient department	n=506	cross-sectional, descriptive study; semi-structured questionnaire	*only 22.5% were found to receive education about safe practices from health care providers<br>*63.2% sleep in a supine position most of the nights<br>*Infants, six months old and younger, were noted to sleep mostly in the same room as their parents (91%).<br>*Infants, six months old and younger, were noted to sleep mostly in the same room as their parents (91%).<br>*supine position was the preferred position for most of the nights in 63.2% of the sample; 22.1% prone & 37% side
Alzahrani et al (2020)[5]	Saudi Arabia; across different regions	2019	To assess Saudi mothers' awareness about SIDS.	Saudi women	n=363	self-reported cross-sectional online electronic survey	*232 (63.9%) of the 363 respondents reported not having heard of any SIDS prevention messages<br>*(53.2%) correctly reported that babies should be laid on their backs; 5.5% of them reported that babies should be laid in their stomachs when being put to rest

Citation	Country	Study period	Study aim	Sample population	Sample size	Design/method	Key findings
Anderson et al (2002)[6]	UK	not reported	To study the context of childcare decision making by inner city and suburban mothers	mothers of normal, full-term newborn babies	n=131	Mixed methods design Individual interview via semi-structured questionnaire at 6-8 weeks after birth followed by six focus groups	▪4% inner city infants & 5.5% suburban infants regularly slept prone<br>▪29% inner city infants & 19% suburban infants occasionally allowed prone sleeping<br>▪58% inner city infants & 55% suburban infants allowed co-sleeping<br>▪At 6-8 weeks 19% inner city infants were BF, 71% fully bottle fed & 10% mixed feeders; 38% suburban infants BF, 33% bottle fed & 29% mixed feeders<br>▪30% inner city mothers and 59% of suburban mothers attempted BF at birth<br>▪52% inner city mothers and 15% suburban mother smoked during pregnancy, a 16% reduction by inner city mothers and 26% by suburban mothers due to pregnancy<br>▪29% inner city mothers and 60% suburban mothers were non-smokers<br>▪74% inner city mothers and 60% suburban mothers consumed alcohol<br>▪93% inner city infants & 98% suburban infants were fully immunised
Anuntaseree et al (2008)[7]	Thailand	Oct2000 – Sep2001	To study bed sharing and sleep position in Thai neonates and the relationship to infant and maternal characteristics	primary care takers with infants 21 days of age	n=3692	Cross-sectional survey design via in home interviews	▪60.6% infants shared a bed with their parents<br>▪39.3% room-shared but in a different bed<br>▪0.14% slept alone in a different room<br>▪60.9% placed their infant supine to sleep, 32.2% side, 4.9% prone, 2.0% no particular way<br>▪Bed sharing and prone sleeping was associated with mothers with a higher maternal socioeconomic status in contrast to previous studies in some Western countries, therefore Cultural differences may play an important role in these findings
Ateah and Hamelin (2008)[8]	Canada; Manitoba	not reported – collected over a 1-month period	To determine mothers' practices and experiences of bedsharing with their infants and also determine their knowledge of the risks.	mothers of infants aged 3-4 months	n=293	Descriptive, cross-sectional survey design via a self-reported questionnaire<br><br>Mailed to 1,122 mothers (26% return rate)	▪42.7% slept with their infants on a regular basis<br>▪29.7% slept with their infants on an occasional basis<br>▪13% indicated the duration they bed-shared was 20min-1hr, 42.5% 2-3hrs, 30% most or all the night<br>▪27.6% never bedshared with their infants<br>▪Majority of mothers agreed that bedsharing had risks for infants, however almost ¾ reported bedsharing on a regular or occasional basis<br>▪74.5% of infants who were bedsharing were breastfed; 53.1% of infants who did not bedshare were breastfed
Austin et al (2017)[9]	US	not reported	To investigate mothers' and fathers' bed-sharing practices and prevention message sources, parent perceptions of source credibility, and the reported effectiveness of these prevention messages to determine the best prevention message source	US community mothers (72%) and fathers (28%) of at least one school-aged child (6-12 years old) from 27 states were recruited	n=678	online survey recruited using a convenience sampling	*47% reported they never bed-shared<br>*28% bed-shared at night<br>*bed-sharing reasons focused on comfort & ease<br>*physicians most common source of advice
Baeis et al (2015)[10]	Iran; Qom	Not reported	To investigate the prevalence of SIDS and the relationship between parental conditions and characteristics with SIDS risk factors.	Parents of infants under 12 months of age in Qom, Iran.	n=1021	cross-sectional descriptive-analytic study, 1,021 infants aged 1 to 12 months in the health centres in Qom-Iran in 2014 were selected as the sample by stratified random sampling method.	• significant association between parental smoking during pregnancy and breastfeeding, in that the number of breastfed infants of non-smoking parents was higher<br>• It is noteworthy that parents' education had a significant association with breastfeeding (P=0.005)<br>• 35.7% bed shared at some point, while 92.3% also room shared<br>• shared bedroom was less common among working parents<br>• No significant association was reported between smoking parents and shared bedrooms<br>• There was a significant association between mother's age and infant sleep position in this study. Additionally, prone or lateral sleep position was more common in infants of working mothers.<br>• Soft pillow was used in 19% of infants. soft pillow was more common among mothers less than 21 years of age

Citation	Country	Study period	Study aim	Sample population	Sample size	Design/method	Key findings
Bailey (2016)[11]	Australia	Not reported	To take an in-depth view of breastfeeding mothers' experiences of bedsharing with infants and young children, in order to better understand why mothers participate in a practice that is outside public policy recommendations and Western cultural practice.	Middle-class, breastfeeding mothers of infants	n=6	Recruitment was purposive through an online Facebook birthing group (four participants) and convenience sampling via personal networks (two participants). Individual semi-structured interviews used to collect data. Qualitative data coded for analysis of recurring themes.	• Reasons for bed sharing centred around the mother-infant dyad getting the best possible night's sleep<br>• The overwhelming response to how bedsharing affected maternal sleep was the increase in both sleep quantity and quality for the mother.<br>• Bed sharing enabled easier feeding throughout the night<br>• Better partner sleep was another common theme brought up by the mothers.<br>• The mothers built communities of friends and other mothers who had similar parenting styles and supported their practice of bed sharing<br>• The mothers demonstrated a good understanding of safe bedsharing practices.<br>• The mothers felt that the breastfeeding experience when bedsharing was strongly enhanced and that breastfeeding and bedsharing were clearly inter-related.
Bailey et al (2020)[12]	Australia	July 2014 - September 2015	To investigate infant sleeping location in a dataset of mothers with strong breastfeeding outcomes. The second aim was to investigate the association between infant sleeping location and breastfeeding. investigate predictors of breastfeeding duration	women who had applied to train as counsellors with the Australian Breastfeeding Association	n=174	A cross-sectional one-group survey design was used. observational design.	• A high proportion of participants in this study bed shared and room shared: At 0–1 month (n= 58), 33% of participants bed-shared, which increased to 58% by 6–12 months (n= 80)<br>• Infants who co-slept were more likely to be exclusively breastfed at 6 months, and had longer breastfeeding duration<br>• Predictors for breastfeeding included maternal education, maternal wellness during pregnancy, birth type, medication during birth, lack of confidence, problems with breastfeeding, lack of knowledge, and conflicting advice
Ball et al (2012a)[13]	UK; Bradford	June2008 – Sep2009	To describe and explore variability in infant care between White British and South Asian families and identify areas for targeted SIDS intervention	families with 2- to 4-month-old singleton infants	n=2560	cross-sectional telephone interviews	▪<3% prone sleep<br>▪29.4% of all infants slept in an infant sleeping bag<br>▪21.1% of White British mothers smoked, a total of 9.8% of all mothers smoked<br>▪18.4% of fathers smoked<br>▪17.8% slept with a pillow, 19.3% with a soft toy and 18.1% under a duvet<br>▪24% White British infants slept in a room alone, compared with <2% Pakistani infants<br>▪9.1% infants ever sofa-shared, with 15.8% White British mothers reporting four times as many than Pakistani infants 4.3% falling asleep on a sofa<br>▪71.5% infants were ever breast-fed, with 36.0% breast-fed for ≥8weeks<br>▪7.7% mothers drank alcohol and 11.4% of fathers drank alcohol<br>▪14.5% swaddled for sleep; 8.6% infants were swaddled when sharing a sleep surface<br>▪44.9% used a dummy at night
Ball et al (2012b)[14]	UK; Bradford	June2008 – Sep2009	To describe the prevalence and associations of bed- and sofa-sharing in a biethnic UK birth cohort	women with infants 2-4 months of age	n=3082	cross-sectional telephone interviews	▪10.2% mothers currently smoked, 19.2% fathers currently smoked<br>▪15.5% families ever bed-shared<br>▪7.2% families regularly bed-shared<br>▪11.6% slept alone in their own room<br>▪43.6% of infants never breastfed or for <1 week, 34.3% infants breastfed ≥8 weeks<br>▪9.4% ever sofa-shared<br>▪1.4% reported both sofa-sharing and bed-sharing<br>▪7.6% of mothers and 11.1% of fathers drank alcohol some evenings or more often
Barbir et al (2020)[15]	Croatia	Data collected in 2018 over two months.	To assess parental knowledge of safe infant sleep and sudden infant death syndrome	Mothers of babies (under 12 months) in Croatia	n=668	46 item questionnaire distributed via social media.	•50% of parents placed baby to sleep on their back, 37% on their side, 14% prone<br>•86% of infants slept with a pillow or stuffed toys<br>•50% room-shared, 41% bed shared
Beck et al (2002)[16]	US	1999	To monitor trends, enhance the understanding of the relations between	mothers of a live infant who was delivered	n=1112 to 2210	Data collected via mailed questionnaire and telephone follow-up	▪ Smoking during the last 3-months of pregnancy ranged from 6.2%-27.2% (sample over 17 states n=1172-2210)<br>▪ Breastfeeding Initiation ranged from 48%-89% (sample over 17 states n=1,131-2,032)

Citation	Country	Study period	Study aim	Sample population	Sample size	Design/method	Key findings
			behaviours and health outcomes, to plan and evaluate programs, to direct policy decisions, and to monitor progress toward Healthy People 2000 and 2010 objectives	in a hospital in the previous 2-4 months with a maximum recall period of 9 months		questionnaire for non-responders	▪ Any breastfeeding for at least 4-weeks after delivery ranged from 34.9%-78.1% (sample over 17 states n=1,131-2,032)<br>▪ Supine sleep positioning ranged from 35.1%-74.6% (sample over 17 states n=1,112-1,998)
Bombard et al (2018)[17]	US	2009-2015	To examine the prevalence of unsafe infant sleep practices	Parents of infants in the US.	not reported. 32 states participated.	Longitudinal survey data. Each US state selects stratified, random sample of women with recent birth. Women surveyed from 2-6 months postpartum using questionnaire. Qualitative data gathered.	•21.6% of parents placed baby to sleep in non-supine position (highest prevalence among African-American community). This decreased from 2009 to 2015.<br>•24.4% of parents often or always bed-shared with infant.<br>•38.5% of respondents used a form of soft bedding
Brenner et al (2003)[18]	US; District of Columbia	Aug 1995 – Sep 1997	To describe sleep practices and the prevalence of bedsharing in a cohort of infants born to predominantly low-income, inner-city mothers, to examine stability in sleep practices during the first 7 to 12 months of life, and identify factors associated with bedsharing.	mothers at delivery and again 3-7 months post-partum	n=394	Prospective birth cohort study via interviews (91% face-to-face). Interviewed at enrolment, shortly after delivery, at 3-7 months post-partum, and at 7-12 months post-partum.	▪90% infants usually slept in the mother's room, 4% usually sleep in their own room<br>▪49% infants slept on an adult bed or mattress the night prior, 43% usually slept on an adult bed or mattress; 0% reported sleeping on a sofa<br>▪40% infants usually sleep with a parent, 47% slept with a parent the night prior<br>▪56% of mothers who smoked bedshared vs 47% of mothers who were non-smokers (p=.15) not statistically significant; 63% of mothers who reported maternal drug use usually bedshared vs 50% of mothers who had no documentation of drug use.<br>▪ 49% of infants who usually bedshare sleep prone
Broussard et al (2012)[19]	US; Florida	2004-2005	To examine the relationship between not using the infant back sleep position and infant bedsharing, identifying maternal characteristics, behaviours and experiences associated with infant sleeping behaviours among non-Hispanic Black and White Florida women	mothers with infants 2-9 months old	n=2791	Data collected via mailed questionnaire and telephone follow-up questionnaire for non-responders	▪42.3% infrequently used supine position<br>▪45.9% frequently bed-shared<br>▪7.6% Black mothers vs 21.4% White mothers smoked after pregnancy<br>▪43.3% Black mothers breastfeed >4weeks vs 54.7% White mothers
Caraballo et al (2016)[20]	US; Colorado	November 2012 - April 2013	To investigate practices, knowledge, attitudes, and beliefs regarding infant sleep among adolescent mothers.	Mothers < 20 years of age completing high school education	n = 43	Adolescent mothers were recruited from high school day-care centres identified through Healthy Child Care Colorado. moderator conducted focus groups. Recordings were transcribed, validated, and deidentified prior to analysis.	• Participants from all groups shared that their sources of parenting information consistently included their mothers, teachers, and medical professionals.<br>• Almost all participants stated that they had learned about SIDS, most commonly from a medical provider or teacher.<br>• When faced with conflicting information, many participants stated they were most likely to listen to their own mothers over medical professionals.<br>• Almost all mothers were aware of and practiced supine sleep.<br>• Most participants were aware of recommendations against using blankets or soft objects in their baby's sleep space.<br>• Most mothers frequently practiced bed sharing.

Citation	Country	Study period	Study aim	Sample population	Sample size	Design/method	Key findings
							• Knowledge of infant safe sleep recommendations was excellent, but adherence with recommendations was poor
Cesar et al (2018)[21]	Brazil; Rio Grande	January 1st 2010 - December 31st, 2010	To know the opinion and identify factors associated with the intention of mothers residing in the city of Rio Grande, to put the baby to sleep belly up.	All mothers who gave birth in Rio Grande during 2010.	n=2395	cross-sectional, survey distributed to mothers within 24 hours of giving birth.	•One-fifth of the mothers showed intention to put the child to sleep belly up, the correct position.<br>• factors associated with this decision were age and mothers' schooling, household income, location of prenatal consultations and number of children<br>•mothers >30 years old are 32% less likely to put their child to sleep in a belly up position than younger mothers<br>•Prevalence ratio for the intention to put the baby to sleep in the supine position was significantly higher among mothers of higher household income and schooling than those with worse socioeconomic status<br>•The higher the parity, the lower the PR for the intention of the mother to put the baby to sleep in the supine position
Cesar et al (2019)[22]	Brazil; Rio Grande	Jan – Dec 2013	To evaluate women's knowledge about the best baby sleeping position and to identify factors associated with a greater probability of putting infants to sleep in an unsafe position	All mothers who gave birth in Rio Grande during 2013.	n=2624	cross-sectional population-based study that included all women who bore children in 2013 in this municipality. A single, standardized questionnaire was given within 48 h after birth. A Chi square test was used for proportions	• 82.1% of mothers stated that the baby should sleep in the lateral or ventral decubitus positions.<br>• 76.4% reported having acquired this knowledge from their mothers<br>• 34.7% were willing to adopt the correct (supine) sleeping position for their child if recommended by doctors<br>• the lower the schooling of the mothers and the greater the number of people per bedroom and number of children, the greater the probability of women choosing an unsafe baby sleeping position.
Chung et al (2003)[23]	US; California	Feb – May 1999	To determine the maternal and infant characteristics associated with the back sleep position for infants to guide efforts to increase its use and reduce the risk of Sudden Infant Death Syndrome	women 10-14 weeks postpartum	n=3349	Cross-sectional self-administered mail survey	▪52.3% infants placed supine; 37.1% side; 10.6% prone<br>▪45.6% of infants who were exposed to smoke during pregnancy were placed side or prone to sleep<br>▪52.5% of infants who were not breastfeeding at 1 month postpartum were placed either side or prone to sleep
Chung-Park (2012)[24]	US	Collection dates not reported - collected over a two month period.	To assess the knowledge, opinions, and practices of infants' sleep positions and their association with demographics.	military beneficiary parents of infants (under 12 months) in the US	n=161	A cross-sectional descriptive design was used to survey a convenience sample of military beneficiary parents being seen at a military treatment facility.	• Forty percent were fully aware of the safe sleep facts for infants; 85% believed supine position was the safest; and 69% of those who believed supine position to be the safest also practiced their belief.<br>• Major reasons for non-supine sleeping positions were for infant preference, comfort, and fear of choking; whereas supine position was for safety reasons.<br>• Parents' opinions of safe position and their practices ($p<0.001$) were significantly associated, whereas knowledge on infant safe sleep facts (p¼0.611) was not.<br>• 87% of parents placed their infant to sleep in a crib or bassinet
Cole et al (2020)[25]	Australia; Queensland	Apr – May 2017	To describe contemporary infant care practices employed by families related to the current public health SUDI prevention program.	primary caregivers with infants approximately 3-months old	n=3341	Cross-sectional self-administered survey via reply-paid post or online	▪only 13% routinely practised all 6 'safe sleep' messages<br>▪34% slept in a non-supine sleep position at some time<br>▪potentially hazardous sleep environments were common with 38% sleeping with soft items/bulky bedding/on soft surfaces<br>▪84% reported baby was smoke-free before and after birth<br>▪room-sharing was usual practice for 75%<br>▪50% shared a sleep surface in the last 2-weeks<br>▪17% no longer received any breastmilk at 8-weeks old

Citation	Country	Study period	Study aim	Sample population	Sample size	Design/method	Key findings
Cole et al (2020)[26]	Australia; Queensland	Apr – May 2017	to establish the maternal and infant characteristics, infant care practices and sleep-related factors that influence breastfeeding duration within a contemporary Australian cohort	caregivers with infants approximately 3-months old	n=3341	Descriptive cross-sectional survey using the Queensland Registry of Births, Deaths and Marriages as sampling frame.	Statistically significant predictors for breastfeeding cessation at 8 weeks include: young maternal age, single, less educated, smoker, public patient, higher pre-pregnancy body mass index (BMI), the birth of a male infant, delivery via caesarean section, in-hospital formula supplementation, admission to a neonatal nursery, dummy use and infant sleep location.
Cole et al (2021) [27]	Australia; Queensland	Apr – May 2017	To investigate primary infant caregiver awareness of the current national public health safe sleep messages and the associations of awareness with care practices.	families with infants approximately 3-months old	n=3341	Cross-sectional survey distributed via post. Responses via either replied paid post or online.	▪96% of families are aware of sleep-related infant mortality and sudden infant death<br>▪26% of caregivers could not identify the current 6-messages in a multi-choice question<br>▪despite being aware of the key messages, some caregiver's practices did not always align with advice (14% were not smoke free, 14% were not usually supine for sleep, 28% employed practices which may increase risk of head/face covering, 18% not receiving breastmilk
Cole et al (2021)[28]	Australia; Queensland	Apr – May 2017	To understand which safe sleep recommendations parents find most challenging to implement, identifying common barriers encountered; and investigate whether challenges are associated with practices employed.	caregivers with young infants approximately 3-months old	n=3341	Cross-sectional survey with qualitative free-text items to explore challenges faced with current recommendations	▪31% of caregivers reported difficulty with at least one safe sleep recommendation.<br>▪Infant sleep position and avoiding bed-sharing were identified as the most challenging recommendations<br>▪families who described difficulty with a recommendation were significantly less likely to consistently employ that advice compared to those who did not report difficulty (sleep position: 198/473,42% vs 2548/2837,90% [p < 0.0001]; own sleep space: (269/344,78% vs 1331/2884,46% [p < 0.0001]).<br>▪Families often proposed alternate strategies with an inference their substitute action compensated potential increased risk when they encountered challenges with a recommendation
Colson et al (2000)[29]	US; Connecticut	June – Oct 1997	To estimate the occurrence of prone sleep among a group of infants in two inner-city clinics and examine the factors associated with parents' choice of sleep position	parents of healthy, term infants at 2-week health supervision visit	n=80	Cross-sectional face-to-face interviews conducted with parents who brought their infants in for their first health supervision visit (convenience sample)	▪31% reported placing their infants prone to sleep some of the time<br>▪Factors influencing prone sleeping: opinion of other people that were regarded as important, worried child would vomit/choke while on their back and prior child-rearing experience<br>▪20% usually slept their child on their back<br>▪Just over 50% parents recall receiving sleep position advice
Colson et al (2001)[30]	US; Connecticut	Dec1999 – Mar2000	To examine the association between the perceptions of inner-city parents about teaching and modelling during the postpartum period of infant sleeping position, and their choice of sleeping position for their infants	parents of infants who were up to 30 days of age	n=100	Cross-sectional survey via face-to-face, semi-structured interviews with a convenience sample	▪42% of infants usually placed supine<br>▪43% usually placed side, 2% usually prone<br>▪26% sometimes placed prone
Colson et al (2006)[31]	US; Boston, Dallas, Los Angeles, New Haven	Jun – Oct 2004	To quantify the degree to which barriers identified in focus groups were associated with the choice of infant sleep position in a group of primarily black, low-income mothers	mothers of infants younger than 8 months	n=671	Cross-sectional survey via face-to-face interviews	▪ 59% usually sleep supine; 25% usually side; 15% prone; 1% other<br>▪ 34% reported ever (meaning usually, sometimes or last night), placed their infant prone
Colson et al (2009)[32]	US; 48 contiguous states	1993 – 2007	To determine trends and factors associated with choice of infant sleeping position	nighttime caregivers of infants born within the last 7 months	n=13,580 (approx.. 1000 interviews each year)	Annual survey via telephone interviews by random sampling	▪ Usual supine position increased to >70% in 2000's compared to 16.9% in 1993<br>▪ 66.8% infants sleep in crib, 9.5% in adult bed

Citation	Country	Study period	Study aim	Sample population	Sample size	Design/method	Key findings
Colson et al (2013)[33]	US	1993-2010	To determine the trends and factors associated with infant bed sharing from1993 through 2010, including the association of physician advice on bed sharing.	Caregivers of infants (<7 months of age)	n=18 986	NISP, cross sectional survey, delivered at random via telephone. Logistic regression used to model trends.	• 11.2% reported an infant sharing a bed as a usual practice.<br>• Bed sharing increased from 1993 (6.5%) to 2010 (13.5%).<br>• factors associated with increase in infant bed sharing as a usual practice included maternal educational level of less than high school compared with college or greater, black or Hispanic compared with White, household income of less than $20000, being born prematurely<br>• 46% of the participants reported talking toa physician about bed sharing<br>• Compared with those who did not receive advice from a physician, those who reported their physicians had a negative attitude were less likely to have the infant share a bed (adjusted odds ratio, 0.66 [95% CI, 0.53-0.82]), whereas a neutral attitude was associated with increased bed sharing (1.38 [1.05-1.80]).
Colson et al (2017)[34]	US	January 2011 - March 2014	To examine the prevalence of both maternal intention regarding infant sleeping position and actual practice and factors associated with their choices	Mothers of infants under six months of age.	n=3297	stratified 2-stage cluster sample design. Multivariable multinomial logistic regression was used to examine associations between measures	• 49.2% mothers place infants exclusively in supine position.<br>• 57.6% mothers intended to place infants exclusively in supine position.<br>• 14.9% of mothers intended to use prone position.<br>• African American mothers were more likely to use prone position.<br>• Those who did not complete secondary education were more likely to use prone position.<br>• Mothers who received advice from doctors that was consistent with AAP recommendations were less likely to place baby in prone position.<br>• Social norms heavily influence practice.
Cornwell et al (2021)[35]	US; Washington DC	Data collected over two and a half months. Dates not reported.	To explore the respective roles of personal social networks and health professionals in affecting new mothers' probability of changing their infant sleep practices over time.	Mothers of infants under six months of age.	n= 323	longitudinal survey data, hospital-based and chain-referral recruitment strategies. Baseline staff-administered survey follow up telephone survey after two and a half months.	•mothers' social networks play a significant role in the likelihood that they adjust their infant sleep<br>practices within the first few months of their infants' lives •Mothers are more likely to change sleep practices<br>when health professionals and/or (lay) family members advise them to do so.<br>•the majority of new mothers engage in safe infant sleep<br>practices from the start, nearly one-quarter of them changed their practices in some way over a three-month follow-up period •most new mothers are not likely to change their infant sleep practices if they initially engaged in safe infant sleep practices from the start. •behaviour change was significantly more likely among those for whom a vast majority of their network members engaged in unsafe infant sleep practices
Corwin et al (2003)[36]	US; Boston, Lawrence, Toledo	Feb 1995 – Dec1998	To describe current newborn sleep practices, to document changes in sleep position with age and calendar time, and to identify determinants and health consequences of various sleep practices among infants during the first 6 months of life.	mothers of newborn infants were enrolled	n=12,029 responded to 1-month follow-up n=11,552 responded to 3-month follow-up	Prospective longitudinal study via mailed questionnaire, non-responders interviewed by telephone	▪ At 1-month prone sleeping declined from 18% (1995-96) to 12% (1997-98); Supine sleeping increased from 18% to 36%; Side sleeping most common with 49% infants (1997-98)<br>▪ At 3-months (1997-98) 52% infants slept supine; 20% placed prone.
Crane and Ball (2016)[37]	UK; Bradford	Not reported	To discover how white British and Pakistani mothers in Bradford recall, understand and interpret SIDS-reduction guidance, and to explore whether and how they implement this guidance in caring for their infants.	mothers of 8 to 12-week-old infants in Bradford	n=46	In-depth narrative interviews with 46 mothers (25 white British origin and 21 Pakistani origin) of 8 to12-week-old infants recruited from the pool of participants enrolled in the 'Born in Bradford' cohort study. Qualitative data gathered.	• All mothers were aware of UK SIDS-reduction guidance (sleep position, surface sharing) from leaflets presented to them during antenatal or postnatal interactions with health care providers<br>• Pakistani mothers tended to dismiss the guidance in toto as being irrelevant to their cultural practices; white British mothers dismissed, adapted and adopted aspects of the guidance to suit their preferred parenting decisions and personal circumstances<br>• Overall none of the mothers interviewed followed all SIDS-reduction guidance and some implemented very little.

Citation	Country	Study period	Study aim	Sample population	Sample size	Design/method	Key findings
Cullen et al (2000)[38]	Ireland	Nov1995 – Mar1996	To examine if Irish parents follow the currently recommended childcare guidelines to reduce the risk of sudden infant death and to examine factors that may have impact on their acceptance	parents with infants in the first year of life were randomly selected from the Birth Register and invited to participate	n=197	Cross-sectional quantitative survey via a semi-structured questionnaire interview	▪54% infant placed supine, 41% side & 1.5% prone<br>▪60% of infants exposed to tobacco smoke in utero<br>▪84% of infants room-shared in the first year of life<br>▪9% slept the entire night and 4% slept >4hours with their parents,<br>▪66% used a dummy during their first year of life, 80% used one daily, 49% from birth and >70% from 2-week-old<br>▪21% of infants used one or more pillows at night
**Cunningham, et al 2018 [39]**	Australia; Victoria	6th October - 17th October 2014	To describe current infant sleeping practices, determining the prevalence and circumstances in which infant bed-sharing is occurring, and identifying the presence of other SUDI risk factors.	Parents of 8-week-old infants who attended a Maternal and Child Health Service.	n=1126	cross-sectional survey of mothers attending the Maternal and Child Health (MCH) Service across Victoria. Data were collected via a self-report questionnaire (42 questions). Descriptive statistical analysis was performed to report the frequency and proportions for each variable.	• Bed-sharing prevalence was found to be 44.7% with 21.5% reporting that this was intended<br>• bed-sharing was less likely amongst those with an annual household income above $AUS104, 000<br>• bed sharing was more likely amongst mothers who breastfed<br>• bassinette was the most frequently reported sleep location (69.7%)<br>• 90.7% had been placed to sleep on their backs since birth<br>• 78.9% of mothers reported that their infant's sleep environment did not include any additional items<br>• mothers born overseas were 1.39 times more likely to bed-share<br>• mothers breastfeeding who were 1.31 times more likely to bed-share
da Silva et al (2019)[40]	Brazil; Pelotas.	January 1st, 2015 - December 31, 2015	To evaluate the prevalence of supine sleep position and its associated factors among 3-month-old infants from a cohort in the city of Pelotas	Mothers of infants born in 2015, in a hospital in Pelotas.	n=4108	evaluated longitudinal data from the 2015 Pelotas Birth Cohort. all mothers of live-born infants in the area were invited to join the cohort. They were then surveyed again at 3 months after birth.	• 55.4% slept in supine position at 3 months and only 66 1.6% in prone position<br>• Maternal white skin colour, higher family income and maternal schooling, advanced maternal age, maternal cohabiting with a partner, receiving counselling from health care professionals and non-bedsharing were associated with higher prevalence of infants sleeping in supine position at 3 months<br>• infant sex, parity and breastfeeding at 3 months were not significantly associated with supine sleep position<br>• mothers older than 18 were from 29 to 65% more likely to adopt supine sleep position compared to younger mothers.<br>• lower prevalence of supine sleep position was associated with black and brown maternal skin colour and bed sharing.
Damato et al (2016)[41]	US	July 2013 - April 2015	To describe safe sleep infant care practices reported by mothers of twins in the first 6 months postpartum	mothers of twin infants	n=35	Mothers caring for twin infants were surveyed online both longitudinally (at 2, 8, 16, and 24 weeks after infant hospital discharge) and cross-sectionally. qualitative data analysed.	• All mothers reported putting both twins supine for all nighttime sleeps and all naps at the postnatal time 1 data point. This decreased over time.<br>• Cribs and play pens/portable cribs were the most common sleep surface for the twins.<br>• The percentage of mothers who reported sleeping the twins on separate sleep surfaces (ie, not sharing cribs) increased over time from 52.2% at postnatal time 1 to 75% at post-natal time 4<br>• Slightly over half of the 6-week-old twins were room sharing at postnatal time 1.<br>• The percentage of twins receiving breast milk decreased over time<br>• Offering a pacifier at time of sleep increased until postnatal time 3<br>• Overall, tobacco exposure was not very frequent in this sample
Douglas et al (2001)[42]	Australia; North Queensland	Oct1997 – Jan1998	To assess awareness of SIDS and the risk reducing recommendations in a sample of mothers in North Queensland, Australia, and to examine their infant care practices	women with young children living who were attending child health care or antenatal clinics	n=195	Cross-sectional interviews using a standardised questionnaire	▪80% slept infant on their back<br>▪85% of infants were breastfed<br>▪55.6% infants occasionally bed-shared, 12.7% always bed-sharing<br>▪29.1% smoked in pregnancy, 7.4% report giving up during pregnancy<br>▪38% report smoking in infant's environment
Duzinski et al (2013)[43]	US; Texas	January 2009 - June 2009	To explore relationship between acculturation and demographic	Hispanic, teen mothers of infants	n=56	Participants were recruited for a cross-	• 38% reported ever bed sharing with their infant.<br>• 27.3% reported the adult bed as the infant or child's primary sleeping space.<br>• Teen mothers with 2 foreign-born parents were more likely to report their infant

Citation	Country	Study period	Study aim	Sample population	Sample size	Design/method	Key findings
			characteristics of Hispanic teen mothers and 3 infant sleep behaviours associated with ASSB risk and protection	completing secondary education		sectional phone survey from 5 high schools.	ever sleeping in an adult bed<br>• Participants who reported living with their boyfriend or husband were less likely to report that their baby ever slept in an adult bed<br>• 89% of participants reported always maintaining a clear sleep space for their infant.<br>• 41.7%) reported not al-ways placing their child in a supine sleep position
Efe and Ak (2012)[44]	Turkey; Antalya	2007; March - May	To determine the Turkish mothers' who have a preterm infant knowledge about risk factors for sudden infant death syndrome (SIDS).	Turkish mothers of preterm infants	n=60	convenience sample of mothers' of preterm infants staying at NICU. Questionnaire (demographic and knowledge questions). Data analysed using SPSS	• 68.3% sharing a room.<br>• 11.7% bedsharing.<br>• 76.7% of mothers planned on using a pillow.<br>• 45.3% of fathers smoked cigarettes.<br>• 63.3% of mothers put their children to sleep in a side position.
Eisenberg et al (2015)[45]	US	January 2011 - February 2013.	To determine whether mothers report receiving advice from doctors, birth hospital nurses, family, and the media about immunization, breastfeeding, sleep position, sleep location, and pacifier use; and to describe the extent to which the advice they report receiving is consistent with current recommendations; and to assess the association between maternal socio demographic factors and maternal report of receiving recommendation consistent advice.	Mothers of infants aged 2-6 months	n=1031	Cross-sectional study. Weighted frequencies of no advice and advice consistent with recommendations were calculated to obtain prevalence estimates. Multivariable logistic regression analyses were performed to assess factors associated with receipt of recommendation consistent advice.	• Although doctors were the most prevalent source of reported advice, ~20% of mothers reported no doctor advice for breastfeeding or sleep position, and more than 50% reported no advice regarding sleep location or pacifier use.<br>• The prevalence of any advice from family or media was 20% to 56% for nearly all care practices, and advice given was often inconsistent with recommendations.<br>• black and Hispanic mothers and first-time mothers were more likely to report recommendation consistent advice
Epstein and Jolly (2009)[46]	UK; England	Nov 2007	To discover why some people in general – but more particularly those in disadvantaged groups – have tended to resist the messages, to help inform future health education strategies	mothers of children aged 6-months to 3-years	n= 506	Cross-sectional study with in-home interview surveys	▪ 14% sometimes slept baby prone; 26% sometimes on the side<br>▪ 24% reported baby slept in same room for 3 months or less<br>▪ 22% reported they sometimes spent the whole night with the baby in their bed while they were asleep<br>▪ over half who took baby into bed to feed, play or comfort, reported they usually did not put baby back in their own cot when the mother wanted to go to sleep
Erdoğan and Turan (2018)[47]	Turkey; Denizli	October 2014 - January 2015.	To identify the risky behaviours of mothers with infants that may put their children at risk for sudden infant death syndrome.	Mothers of infants < 12 months of age	n=456	Cross-sectional, descriptive study. Questionnaire administered via face-to-face interview. Logistic regression analysis was performed to determine the relationship between sleep position and potentially risky behaviours of mothers	• The greatest risk factor is the infant's sleeping position.<br>• A total of 77.9% of the mothers put their babies in bed in a non-supine position<br>• 65.8% used a pillow when they put their babies in bed,<br>• 52.9% used a soft mattress<br>• 28.5% shared their beds with their babies.<br>• Prone sleeping was more likely to occur when smoke was present in the home or a pillow was used.

Citation	Country	Study period	Study aim	Sample population	Sample size	Design/method	Key findings
Erick-Peleti et al (2007)[48]	New Zealand; Auckland	Mar – Dec 2000	To describe the association between cigarette smoking and maternal factors amongst a cohort of Pacific mothers	mothers of Pacific infants born in Middlemore Hospital	n=1,219	Prospective cohort study design. Interviews at 6-weeks and 12 months postpartum	▪ 24.5% mothers smoked at 6 weeks<br>▪ 29.8% mothers smoked at 12 months<br>▪ At 12 months 9.6% mothers had started smoking since the 6 weeks postpartum interview compared to 4.4% who had stopped<br>▪ 20.2% mothers were smoking at 6-week and 12-months postpartum
Fernandes et al (2020)[49]	Portugal	30 June - 27 September, 2019	To conduct a survey with parents and healthcare professionals at the Centro Hospitalar Universitário São João (CHUSJ), in order to assess their knowledge of SIDS and its risk factors	Parents of infants and healthcare professionals in obstetric and neonatal care departments	n=204 parents n=113 healthcare professionals	Structured, self-administered questionnaires delivered to parents in the obstetrics and neonatology ward. collected data on the respondents' self-perception of their knowledge about SIDS, and demographic background. performed a descriptive analysis of the responses, and logistic regressions and odds ratios were used in order to investigate the relationship between variables.	• 100% of healthcare professionals and 67.7% of parents were aware of SIDS<br>• 82.3% of healthcare professionals and 47.5% of parents recognized the supine position as the safest to prevent SIDS<br>• only 37.2% of healthcare professionals responded correctly to 75% or more of the 13 questions about SIDS' risk factors.<br>• among parents, only seven questions (out of 13) were correctly answered by the majority of respondents<br>• Parents declared that their main sources of information about SIDS were the Internet (53.7%), nurses (34.5%), and doctors (25.9%).<br>• vast majority of parents rated their knowledge about SIDS and its risk factors as moderate (42.5%) or (very) low (43.9%)<br>• majority of parents also described their confidence to discuss SIDS-related issues as moderate (39.6%) or (very) low (47.5%)<br>• Healthcare professionals rated their knowledge level about SIDS between high or very high (30.1%), moderate (48.7%), and low or very low (21.2%)<br>• Healthcare professionals confidence to discuss SIDS-related issues was rated high or very high in 21.2% of the cases, moderate (46.9%), and low or very low (31.9%).<br>• 6.9% of healthcare professionals reported giving recommendations to parents about SIDS prevention more than once a week, while only 53.1% of them considered themselves as qualified to advise parents about SIDS and its risk factors<br>• doctors were more likely to have less correct knowledge about SIDS risk factors than nurses, and also to give worse recommendations about the safest sleep position
Ford et al (2000)[50]	New Zealand; Canterbury	May 1997	To describe current prevalence of infant sleep practices related to SIDS risks	mothers of 8-week old infants	n=274	Cross-sectional survey via mailed questionnaire<br>▪ Compared results to the control figures derived from the New Zealand Cot Death case-control Study (NZCDS) data collected 1987-90 n=174 eligible control infants	▪ 51% infants shared a room with their mother<br>▪ 43% slept in a room alone<br>▪ Of infants usually room-sharing 75.9% in 1997 slept in their own bed compared to 48.9% in the NZCDS<br>▪ 2.9% infants regularly slept prone in 1997 compared to 39.7% in NZCDS<br>▪ Sheepskin use declined with 30.7% in 1997 compared to 46.2% in NZCDS
Fowler et al (2013)[51]	US; Texas	January - July 2011	To compare parents of infants cared for in newborn intensive care units (NICUs) and infants cared for in well-baby ("general") nurseries with regard to knowledge and practice of safe sleep practices/ sudden infant death syndrome risk reduction measures and guidelines. Also, to obtain qualitative data regarding reasons for noncompliance in both populations.	Caregivers of infants <12 months old	n=60	Cross-sectional survey. Caregivers surveyed during first/second postnatal visit. Survey administered measuring knowledge and practice. Participants recruited from UT Kids' Place Clinic or High-Risk Infant Clinic.	• NICU parents were scored higher on the knowledge section of the survey<br>• NICU parents had more "perfect sleep practice" than parents of WBN infants<br>• There was also a trend toward NICU parents practicing less bed sharing than WBN parents<br>• There was a positive association between knowledge and practice of SIDS risk reduction measures<br>• most common reasons for not following the SIDS risk reduction guidelines was "parental preference." (ie. they felt safer having the baby in the bed with them so they could monitor them closely)<br>• A small number of parents practiced bed sharing because they did not have a crib

Citation	Country	Study period	Study aim	Sample population	Sample size	Design/method	Key findings
Galland et al (2014)[52]	New Zealand; Dunedin	June 2009 - February 2011	To describe childcare practices related to SUDI prevention messages in a New Zealand community, and to develop and explore the utility of a risk assessment instrument based on international guidelines and evidence.	Parents of infants born between June 2009 and February 2011	n=209	Prospective longitudinal study recruited antenatally. Participant characteristics and infant care data collected by questionnaire at: baseline (third trimester), and monthly from infant age 3 weeks through 23 weeks. Published meta-analyses data were used to estimate individual risk ratios for 6 important SUDI risk factors which, when combined, yielded a "SUDI risk score".	▪ Groups within the community identified as priorities for education about safe sleep practices beyond standard care are mothers who are young, have high parity, low educational levels, and have symptoms of depression<br>▪ Supine sleeping was practiced by 90% breastfeeding by 89%, and not bed sharing in combination with no maternal smoking by 81%. ▪The only recommended practice that few parents followed was regular use of a pacifier (19%).<br>▪ The SUDI risk score could range from a possible 1.0 (avoiding all risk factors) to 160.6 (with all risk factors present). The arithmetic mean score in this study was 3.1 with a median of 1.4 (values ranged from 1.0-61.0).<br>▪ the main safe sleep messages for SUDI prevention are highly practiced within this NZ community sample.
Gaydos et al (2015)[53]	US; Georgia	Not reported	To compare decision making regarding sleep practices for low-income, African-American women and counselling practices of their providers to better understand how to effectively mitigate SIDS/SUID risk for this population.	African-American, low income, first-time mothers of infants < 6 months of age	n=60	Eight focus groups with mothers. 20 interviews with medical providers via telephone. Participants recruited via flyer distribution in waiting rooms at public health centres. Qualitative data collected. Coded for analysis of themes.	• Large majority of participants were aware of and understood safe sleeping recommendations<br>• all but three of the African American women reported bed sharing with their infants<br>• reasons for bed sharing included perceived safety, convenience, improved infant sleep quality<br>• Providers recognized that many mothers are noncompliant and attribute non-compliance largely to cultural and familial influence<br>• few provider attempts are made to mitigate SIDS risks from non-compliant behaviours
Gibson et al (2000)[54]	US; Philadelphia	Dec 1995 – Feb1997	To compare sleep position practices and other SIDS risk factors among a variety of groups and examine reasons given for current practices	parents of infants 6 months of age or less	n=410	Cross-sectional survey via interview questionnaires	▪ 15% mothers smoked during pregnancy; 17% smoked at time of survey; 34% had at least one household members smoking<br>▪ 71% reported bottle-feeding infants with 31% reporting breastfeeding<br>▪ 23% regularly co-slept in an adult bed; 46% occasionally co-slept in an adult bed<br>▪ 19% occasionally slept on a couch<br>▪ 72.7% reported placing infants in the non-prone position<br>Disparity between some demographic groups persists. The majority of parents placing their infants prone report they do so because their infant is more comfortable or sleeps better despite 73% saying their physician/nurse had discussed sleep position with them.
Goodstein et al (2015)[55]	US; Pennsylvania, Maryland	August 2010 - March 2012	To evaluate a comprehensive hospital-based infant sleep safety (ISS) program on parental education and safe sleep behaviours in the home.	Caregivers of infants	n = 1,582	This study tested the effectiveness of a system-level intervention. Cross-sectional survey of parents of healthy newborns at the time of hospital discharge (HD) and at 4-month well-childcare visit (follow-up). Participants were recruited in the hospital by maternity nursing staff. Data were analysed by chi-square	• supine sleep knowledge was 99.8% at HD. 4.8% of families planned to always use this position<br>• At F/U, 97.3% retained supine knowledge, and 84.9% maintained this position exclusively<br>• Knowledge of crib as safest surface was 99.8% at HD and 99.5% F/U<br>• Room sharing with fell to 91.9% (HD) and 68.2% (F/U)<br>• the F/U group was more likely to use supine positioning and a bassinette or crib.<br>• Knowledge of pacifier use being protective against SIDS increased from 57.2% (HD) to 65.5 (F/U)<br>• Families felt that they received the correct amount of ISS education (97.3%, HD; 89.6%, F/U)<br>• intention to follow safe sleep practices and use of actual safe sleep practices were higher in parents receiving ISS education

Citation	Country	Study period	Study aim	Sample population	Sample size	Design/method	Key findings
						and z test of proportions.	
Haas et al (2017)[56]	US	Not reported	To determine the extent to which the infant care practices of mothers of higher-order multiples adhere to current AAP SIDS recommendations over the infants' first year of life	mothers caring for their first set of higher-order multiples < 12 months	n=14	Mothers caring for higher-order multiple-birth infants were recruited from an online support group. An online survey was used to assess infant care practices when the infants were first brought home from the hospital as well as at the time of the survey. Descriptive analysis of results.	• Nighttime supine sleep fell from 78.6% adherence at HD to 64.3% at time of survey<br>• Room sharing fell from 50% to 21.4%<br>• no bed sharing increased from 71.4% to 92.9%<br>• breastfeeding decreased from 78.6% to 35.7%<br>• pacifier use fell from 28.6 to 21.4%<br>• the use of blankets and toys in cribs increased after HD
Hamadneh et al (2016)[57]	Jordan	winter season of 2015/ 2016	To evaluate maternal common practice for infant sleep care and the home environment, in order to explore the major risk factors associated with sudden unexpected infant death in Jordan.	mothers with infants <1 year old	n= 604	Data were collected via semi-structured questionnaire interview. he information questionnaire was filled out during face-to-face interview with the principal investigator. Descriptive statistics were generated.	• 92% indicated that they do not put infants in a prone position to sleep. 90% slept in their parents' room at night.<br>• 55% of mothers indicated using wedged objects, such as bumper pads and pillows near the infant during sleep.<br>• 67% of the mothers indicated using blankets.<br>• 84% of mothers covered the infant's head.<br>• 56% occasionally shared their beds with their infants for more warmth. 73% of mothers used wood, kerosene or gas heaters in the baby's room with closed windows to keep the room warm.<br>• 71% of mothers indicated that their infants were exposed to tobacco smoke
Hannan et al (2020)[58]	US; Colorado	2000-2013	To evaluate the association between neonatal intensive care unit (NICU) admission and breastfeeding practices, infant supine sleep positioning, and postnatal smoking among mothers of late preterm infants.	Mothers of late preterm infants	n=62 494	Population based study. Data from 36 states using the 2000-2013 PRAMS were analysed. chi squared tests and 95% CI assessed infant and maternal characteristics and recommended care practices for late preterm infants based on NICU admission after birth.	• 48.7% spent time in a NICU<br>• mothers of late preterm infants hospitalized in the NICU were more likely to initiate breastfeeding than were mothers of late preterm infants not hospitalized in the NICU<br>• mothers of NICU late preterm infants were more likely to put their infant to sleep in a supine position<br>• There was no difference between groups for postnatal smoking
Hauck et al (2008)[59]	US; a nationally distributed consumer opinion panel of >500,000 households	May2005-Jun2007	To examine the sleeping arrangements for infants from birth to 1 year of age and to assess the association between such arrangements and maternal characteristics	mothers of infants 3-12months old	n=approx. 2300 at 3-months n=approx. 1800 at 12-months	Longitudinal study via telephone and mailed questionnaires. Collected data analysed from the 3-, 6-, 9-, & 12-month questionnaires	▪ 71%-79% 2-weeks to 6-months were placed supine<br>▪ 12%-17% 2-weeks to 6-months were placed prone<br>▪ 5%-16% 2-weeks to 6-months were placed side<br>▪ 45.1%-85.3% 2-weeks to 6-months slept in mother's room at night<br>▪ 30.7%-42.5% 2-week to 6-months bedshare<br>▪ 6.1%-17.8% of infants sleep/lie down with their mothers on a couch<br>▪ 2.5%-3.1% infants sleep in an attached co-sleeper
Hauck et al (2015)[60]	US; Washington DC, Indiana	2010–2011	To describe parental knowledge and practices regarding infant sleep position, bedsharing, pacifier use, and feeding practices before and after	Low income families with an infant	n=4786	Longitudinal study. Parents completed surveys before (''prenatal'' and ''postnatal'') and 1–3 months after crib and	• 76% percent of mothers who completed the survey prenatally and 77% postnatally stated correctly that the recommended sleep position for healthy babies was on their back. After the intervention, this increased significantly to 94 % of mothers stating that the recommended sleep position was on the baby's back<br>• Only 8 % of parents intended to bedshare when asked prenatally, while 38 % had bedshared the prior night when asked postnatally. After the intervention, bedsharing rates

Citation	Country	Study period	Study aim	Sample population	Sample size	Design/method	Key findings
			receipt of a free crib and safe sleep education.			safe sleep training receipt (''follow-up''). Descriptive and bivariate analyses were completed.	decreased to 16 %<br>• Ninety percent reported that the baby slept in a crib after the intervention, compared with51 % postnatally<br>• Parental knowledge of recommended infant sleep position improved from 76 % (prenatal) and77 % (postnatal) to 94 % after crib receipt
Hirabayashi et al (2016)[61]	Japan;	July 2010 - March 2011	To assess parental knowledge and current practices of environmental factors related to SIDS and to rebuild strategies for resuming a continued decrease in the prevalence of SIDS in Japan.	Parents of infants under 12 months residing in Japan.	n=4319	Parents were invited to participate in the study at discharge from the maternity hospitals. Participants answered a questionnaire at their one month check-up, and again at one year. Qualitative data assessed using chi-squared test and logistic regression.	• 96.7 % answered that they lay their infants to sleep in the supine position exclusively.<br>• 81.4 % of parents were aware of the infant prone position being a SIDS risk factor.<br>• Parental awareness of smoke exposure as a risk factor was 69.0 %.<br>• awareness of breastfeeding having a protective effect was 47.8 %.<br>• paternal smoking habits had decreased from the one-month medical check-up (39.5 %) to one year later (37.2 %).<br>• maternal smoking significantly increased from 1.5 % to 3.9 %. 92.7 % of parents fed infants by either breast or mixed feeding.
Homer et al (2012)[62]	Australia	Mar 2010	To examine sleep patterns and strategies including bed-sharing.	Women aged over 18 who have infants aged up to 24 months	n=1000	A descriptive study was undertaken using an online, anonymous questionnaire. Cross sectional, qualitative data. Simple descriptive statistics were calculated.	• 92% of respondents reported difficulties with getting their infants to sleep<br>• 97% felt sleep-deprived at some time with almost half reporting that they were always or regularly deprived of sleep.<br>• Strategies to facilitate infant sleeping included rocking and patting (50%), giving a dummy/comforter (46%) and allowing the baby to fall asleep in their arms (47%) or after feeding (45%).<br>• Just under half (41%) utilised bed-sharing as a sleep strategy at night.<br>• Bed-sharing was more likely to be used if babies experienced frequent waking at night and unstable sleep patterns.
Hussain et al (2018)[63]	US; Illinois	October 1, 2016 - May 18, 2017	To describe and analyse female caregivers served by Illinois MIECHV-supported home visiting programs to determine and compare their safe sleep behaviours by breast feeding status and tobacco use.	Mothers of infants (<12 months) in at-risk communities.	n=289	Cross sectional survey. Participants were mothers enrolled in an IECHV-home visiting program. Caregivers responses to three safe sleep questions were compared by breastfeeding status, caregiver tobacco use, and household tobacco use using Pearson's chi-squared or Fisher's exact test.	• 85% caregivers always placed infants in the supine position,<br>• 51% never bed-shared<br>• 64% never used soft bedding<br>• Ongoing breastfeeding caregivers never bed-shared more often than those who never breastfed or weaned (66%vs. 53% vs. 39%)<br>• Households with tobacco use placed infants in the supine position less (75% vs. 88%), bed-shared more (62% vs. 44%), and used soft bedding more (50% vs. 32%)
Hutchison et al (2006)[64]	New Zealand; Auckland	Apr – May 2005	To survey the knowledge and implementation of sudden infant death syndrome (SIDS)-protective infant care practices in mothers of infants aged less than 4 months	mothers with infants aged 6-8 weeks [n=143] and 3-4 months [n=135]	n=278	Cross-sectional design via postal questionnaire with reminder phone call at 2-3weeks for non-responders	▪ 72% supine, 14% side, 12% side and back and 1% prone last night<br>▪ 65% supine, 10% side, 22% side or back and 3% prone as the usual position<br>▪ smoking during pregnancy nearly 8% mothers; 7% reported smoking in last 24hours<br>▪ 97% ever breastfed; 79.4% of all infants BF in last 24hrs, 74% of 3-4 months BG in last 24hrs<br>▪31% infants usually used a dummy<br>▪ 54% usually slept in the same room as their parents; 39% infants usually slept in their own bed in the parent's room<br>▪16% infants co-slept for 2 or more hours<br>▪46% usually slept in their own room<br>The benefits of supine sleeping, not smoking and breastfeeding appear to be well understood by respondents. More education is needed about other SIDS-protective

Citation	Country	Study period	Study aim	Sample population	Sample size	Design/method	Key findings
							behaviours such as keeping the face clear and sleeping infants in their own beds in the parents room.
Hutchison et al (2007)[65]	New Zealand; Auckland	Apr – May 2005	To survey current infant sleep position practices, concerns about plagiocephaly and the use of sleep positioning devices	mothers with infants aged 6-8 weeks [n=143] and 3-4 months [n=135]	n=278	Cross-sectional design via postal questionnaire with reminder phone call at 2-3weeks for non-responders	▪ 64.8% usually placed supine<br>▪ 2.9% reported usually using prone with 3.7% of the 3-4months usually prone<br>▪ 30% of 3-4 month olds used positioning devices during sleep, with 34% of 6-8 week olds<br>▪ 18% used foam wedge devices; 11% used a Safe T Sleep Sleepwrap (a fabric sheet that wraps around the mattress with an attached portion wrapped about the infants' abdomen to maintain sleeping position); 5% used a rolled towel or blanket or similar tucked behind/beside the infant; 5% used more than one device
Hutchison et al (2010)[66]	New Zealand; Auckland [infants born at National Women's Hospital Auckland]	Oct 2007 – Feb2009	To determine the prevalence of co-bedding, infant care practices related to sudden infant death syndrome (SIDS), and the mother's knowledge of SIDS risk factors	mothers of live-born twins born at National Women's Hospital, Auckland	n=109 [at 6-weeks]<br>n=91 [at 4-months]<br>n=90 [at 8-months]	Cross-sectional study via postal survey	▪ 4.6% mothers smoked in pregnancy<br>▪ At 6-weeks - 75.9% usually placed supine, 10.2% usually side, 0% prone; at 4-months – 85.7% usually supine, 8.8% usually side, 3.3% prone, 4.4% varied; at 8-months – 73.3% supine, 13.3% side, 5.6% prone, 7.8% varied<br>▪ 83% still breastfeeding at 6-weeks, 61% at 4-months, 31% at 8-months<br>▪ 45% 6-week, 34% 4-month, 27% 8-month were room-sharing in the parents room<br>▪ 28% 6-week usually bedshared with their twin in the parents; 13% 6-week slept in own bed in parents room<br>▪ 38% 6-week usually used a dummy; 50% at 4-months; 38% 8-months<br>▪ 52% 6-week usually co-bedded with twin; 31% 4-months; 10% 8-months<br>Cobedding of twins was very popular, especially at 6-weeks of age. Further research is needed to establish the safety of cobedding in relation to the risk of SIDS
Hutchison et al (2015)[67]	New Zealand; Auckland	Not reported	To evaluate mothers' knowledge of, and practices related to, risk factors for sudden unexpected death in infancy (SUDI) and to compare results with a similar survey conducted in 2005.	women with infants between the ages of 6 weeks and 4 months	n=172	randomly selected women who had delivered an infant at National Women's Health hospital were sent a postal questionnaire asking about their knowledge and infant care practices related to SUDI risk factors. cross sectional survey. Chi-squared analysis was used for categorical variables, and t-tests for continuous variables.	• Compared with 2005, more women in this survey cited avoiding bed sharing, keeping the face clear, avoiding soft bedding, and room sharing as SUDI prevention factors.<br>• compared with 2005, more mothers usually used the supine sleep position and shared the parental bedroom, while fewer mothers reported smoking.<br>• 8% percent said the infant usually shared a bed, down from 15% in 2005.<br>• Of the five main protective factors promoted by New Zealand's Ministry of Health (supine sleep, own bed, room sharing, smoke free, breastfeeding), 43% were implementing all of these practices.<br>• Only four mothers (2%) had smoked in pregnancy, down from 8% in 2005<br>• Pacifiers were usually used by 29%, similar to the 31% using them in the earlier survey.
Hwang et al (2013)[68]	US	2000-2008	To compare the timing of hospital discharge, time to outpatient follow-up, and home care practices for late-preterm and early term infants with term infants.	Mothers of infants in the US	n=242271	Analysed PRAMS data.	• Late pre-term infants were less likely to be discharged early<br>• LPT and ET infants were more likely to have timely outpatient follow-up, more likely to experience maternal tobacco smoke exposure, less likely to be initially breastfed, and less likely to be placed in a supine sleep position than term infants
Hwang et al (2016)[69]	US	2000-2011	To compare the prevalence of SSP after hospital discharge for preterm and term infants in the US; and to assess racial/ethnic disparities in SSP for preterm and term infants	Mothers of infants in the US	n=392,397	Analysed PRAMS data from 2000-2011. measured prevalence of SSP by preterm and term gestational age (GA) categories. calculated adjusted prevalence ratios (APR) to evaluate the	• Prevalence of SSP varied by GA<br>• late preterm infants were slightly less likely to be placed in SSP compared with term infants<br>• There were racial/ethnic disparities in SSP for all GA categories when Non-Hispanic-Black and Hispanic infants were compared with Non-Hispanic-White infants<br>• NHB and Hispanic infants had significantly lower prevalence of SSP compared with NHW infants<br>• Overall, prevalence of SSP was 66.5%

Citation	Country	Study period	Study aim	Sample population	Sample size	Design/method	Key findings
						likelihood of SSP for each GA category compared with term infants and the likelihood of SSP for non-Hispanic black (NHB) and Hispanic infants compared with non-Hispanic white (NHW) infants.	• Infants born at ⩽ 27 weeks had the lowest prevalence of SSP compared with all other GA groups
Inbar et al (2005)[70]	Israel; Haifa District	15 -21 July 2001	To study parents' compliance with the recommendation to avoid placing healthy infants to sleep in the prone position and to identify characteristics of noncompliant parents of infants aged less than 6-months-old	parents of infants aged 0-12 month who visited the primary preventive health centres	n=1,912	Cross-sectional study via self-administered questionnaire	▪Infants aged 0-3 months - 30.6% were placed supine, 12.4% prone, 38.3% side, 13.5% inconsistent & 5.1% side and supine<br>▪Infants 3-6 months- 36.4% supine, 17.6% prone, 28.1% side, 13.9% inconsistent & 3.9 side and supine
Joyner et al (2016)[71]	US; Washington DC, and Maryland.	Between July 2006 and December 2008	To investigate African-American parental reasons for pacifier use or non-use, and whether knowledge of the association with decreased SIDS risk changes decisions about pacifier use.	African-American parents of infants (0-6 months).	n=83	Cross-sectional sample. Qualitative analysis software used to code data. Focus groups and individual interviews.	• 72.3 % of infants used pacifiers.<br>• Reasons for pacifier use included comfort/soothing, safety/SIDS, and preference over digit sucking.<br>• Reasons for pacifier non-use included infant refusal, fear of attachment, nipple confusion, and germs.
Kelmanson (2013)[72]	Russia	Not reported	To assess the association between swaddling and sleep behaviour in two-month-old infants, the age known for high risk of SIDS.	Mothers of infants around 2 months of age	n=198	Random sampling method. Cross-sectional questionnaire. Statistical analysis was based on the chi-square test	• (60.6%) of babies were never swaddled at night, 17.7%) were swaddled occasionally, (21.7%) were swaddled regularly (every night)<br>• mothers who occasionally swaddled their infants were characterised as having lower educational level, more often living in a restrained household, and more often being unmarried.<br>• the majority of babies were put to sleep either on their back or on their side<br>• non-swaddled infants were more likely to be put to sleep in supine position<br>• swaddled infants were more likely to be put to sleep with a pacifier<br>• swaddled infants were more likely to be put to sleep on a soft mattress
Kihlström et al (2020)[73]	US; Florida	Not reported	To explore barriers, facilitators, and home visitor influences on infant sleep practice	Caregivers of infants <6 months residing in Florida.	n=5	Cross-sectional sample. Participants recruited at random. An instructional sheet was mailed to participants, using simple language and graphics. Participants reflected on each of the three statements. A phone interview was conducted after completion of the survey for clarity. Qualitative analysis included thematic analysis.	• furnishing, knowledge, routines, and social support were the main facilitators contributing to safe sleep practices<br>• Visual reminders (e.g. safe sleep stickers) were also mentioned.<br>• The main barriers to safe sleep practices were found to be housing issues, other caregivers' practices, and influence of siblings.<br>• the home visitor's role went beyond safe sleep training as they were considered trust-worthy allies who provided a wide range of information, resources, and support.<br>• All participants described that having prior knowledge of safe sleep practices guided their decisions regarding infant sleep practices at home.<br>• Participants' pictures demonstrated that they were aware that no soft bedding, pillows, or toys should be placed in the crib with the child ,as is recommended by the AAP

Citation	Country	Study period	Study aim	Sample population	Sample size	Design/method	Key findings
Konstat-Korzenny et al (2019)[74]	Mexico	1st May - July 30th 2019	To assess the sleeping habits of infants in their homes, and parental compliance with the AAP safe sleep guidelines.	Caregivers of infants <12 months old	n=184	cross-sectional study. Subjects were selected via convenience sampling at one hospital in Mexico. Data collected via verbal questionnaire.	• 78.8% of participants practiced bed sharing<br>• 48.9% of infants slept supine<br>• 2.7% of infants used a pacifier<br>• no patients followed all the recommendations in the questionnaire<br>• over 10% followed no recommendations at all<br>• 64% of respondents used a pillow to put their child to sleep
Krouse et al (2012)[75]	US; Pennsylvania	Not reported	To examine the infant bed-sharing practices of mothers from the birth of the infant to three months of age	Mothers of newborn infants	n=94	a longitudinal descriptive design using a self-report instrument immediately after delivery with follow-up phone interviews at one and three months after discharge. Self-reporting method used to obtain data. Analysis of the quantitative data included the use of descriptive statistics and t-tests. Qualitative data were analysed using line by line coding which was then reviewed and categorized.	• While no mothers intended to bed-share with their infants immediately after delivery, 47 percent reported bed-sharing at some time at one month after discharge and 9 percent at three months<br>• Only 19 percent of mothers reported receiving information about infant sleeping practices from their physician and 22 percent from their nurse<br>• One month post discharge was identified as a high-risk period for infant bedsharing.
Kuhlmann et al (2016)[76]	US	April - August 2014	To partner with obstetrical providers to increase promotion of the American Academy of Pediatrics guidelines for infant safe sleep. Specifically, evaluate the effectiveness of the Safe Sleep Toolkit during obstetrical visits. Secondary objectives include improving provider and maternal knowledge of safe sleep.	Pregnant woman (between 24-36 weeks), and obstetrical providers	Pregnant women (n = 111) (56 pre-intervention and 56 post-intervention)	Study conducted at a private obstetrical group practice. Surveys administered by a research assistant to mothers following appointments. The survey included 14 questions regarding: (1) knowledge (2) intention (3) sources of knowledge/education, (4) whether recommendations were discussed at visit. and (5) sources of inconsistent messages. The intervention consisted of training in the Safe Sleep Toolkit. One month post intervention, surveys were readministered, and results were compared. Quantitive analysis.	• Significantly more post-intervention women reported their provider had discussed safe sleep (78%vs 32%)<br>• Similarly, provider-reported discussion with women increased significantly for all safe sleep guidelines (82%-90% vs 8%-12%)<br>• Maternal knowledge, especially surrounding unsafe sleep practices, improved significantly post-intervention.<br>• Other sources of safe sleep knowledge included print media (55%), family or friends (45%), hospital staff (34%), online sources (30%), local programs (23%), television (20%), obstetrics provider/staff at previous visit (20%), and different doctor/staff (11%)<br>• Pre-intervention, 11% of women were able to identify all 19 safe sleep elements. Post-intervention, this increased to 31%<br>• intention to put an infant to sleep with blankets reduced from 32% to 13% post-intervention<br>• Health providers reported barriers to providing safe sleep anticipatory guidance for women included lack of familiarity with guidelines (n = 6,55%), lack of time (n = 8, 73%), belief that safe sleep discussion was family practice or paediatric providers' responsibility (n = 6, 55%), and lack of structured process (n = 6, 55%).

Citation	Country	Study period	Study aim	Sample population	Sample size	Design/method	Key findings
Lahr et al (2005a)[77]	US; Oregon	Nov 1998-Oct1999	To explore the relationship between maternal smoking and bedsharing among Oregon mothers to explore whether smoking mothers, in contrast to non-smoking mothers, are getting the message that they should not bedshare	mothers of a live infant who was delivered in a hospital in the previous 2-4 months with a maximum recall period of 9 months	n=1756	Mixed-mode data collection method via mailed questionnaires and telephone follow-up for non-respondents	▪ 20.5% reported always bedsharing; 14.7% almost always; 41.4% sometimes; 23.4% never<br>▪ Bedsharing of smoking mothers 18.8% always; 12.6% almost always; 45.1% sometimes; 23.6% never (not statistically different from non-smoking mothers)
Lahr et al (2005b)[78]	US; Oregon	1998- 1999	To identify groups of Oregon women who were most likely to put their infant to sleep prone	mothers of a live infant who was delivered in a hospital in the previous 2-4 months with a maximum recall period of 9 months	n=1732	Mixed-mode data collection method via mailed questionnaires and telephone follow-up for non-respondents	▪ 9.2% usually slept prone; 24.2% side; 66.5% supine
Lahr et al (2007)[79]	US; Oregon	1998- 1999	To explore the prevalence and determinants of bedsharing in Oregon	mothers of a live infant who was delivered in a hospital in the previous 2-4 months	n=1756	Mixed-mode data collection method via mailed questionnaires and telephone follow-up for non-respondents	▪ 20.5% reported always bedsharing; 14.7% almost always; 41.4% sometimes; 23.4% never<br>▪ Breastfed >4 weeks 75.4% ≤4 weeks 24.6%<br>▪ 20.1% Smoking postpartum<br>▪ 67% usually place infant supine, 24.0% side, 9.0% prone
Lau and Hall (2016)[80]	Canada; Vancouver	December 2012 - July 2013	To explore Canadian mothers' experiences with infant sleep safety.	Primary caregivers of infants<6 months	n=14	Purposeful sampling via promotional posters in community centres and libraries. Semi-structured interviews and constant comparative, qualitative analysis. The first author conducted face-to-face, semi-structured interviews with each participant.	• Mothers' experiences were influenced by four factors: perceptions of everyone's needs, familial influences, attitudes and judgments from outsiders and resource availability and accessibility<br>• All mothers identified back to sleep in a crib or bassinette as the safest approach.<br>• Mothers found disparities between their visions of sleep safety, and the reality of their infants sleeping pattern.<br>• Mothers acknowledged that their modified sleep arrangements increased the risk of SIDS.<br>• Mothers viewed deviating from the safe sleep recommendations as acceptable because they were generally following safe sleep principles
Martiniuk et al (2016)[81]	Australia; Sydney	January - November 2014	To better understand families' experiences of plagiocephaly by systematically exploring perspectives of the expecting parent of a first or subsequent child, parents with a child or children, grandparents and clinicians	parents/grandparents of children with plagiocephaly and clinicians who treat plagiocephaly	n=121	Cross-sectional study. Participants were selected with a combination of purposive sampling and snowball sampling. Semi-structured interviews/focus groups to gather qualitative data. Interviews coded for analysis of themes.	• Plagiocephaly worried most parents because it could permanently affect their child's 'looks' and some thought it would affect a child's development.<br>• Parents were 'willing to do anything 'to prevent plagiocephaly including using products or sleeping positions that are contraindicated under sudden infant death syndrome guidelines<br>• Parents found the care pathway convoluted and inconsistent messages were given from different health providers.

Citation	Country	Study period	Study aim	Sample population	Sample size	Design/method	Key findings
Mathews et al (2015)[82]	US; Washington DC, and Maryland.	July 2006 - July 2012	To compare infant care practices pertinent to sleep-related infant deaths, including sleep position, bedsharing, room-sharing, parental smoking and breastfeeding, among African–American and Hispanic families	African–American and Hispanic mothers of infants<6 months of age	n=512	Cross-sectional, Multimodal study. Gathered qualitative and quantitative data via surveys, interviews, and focus groups.	• African–American infants were more likely to be placed prone, share the bed with the parent, and to be exposed to smoke<br>• Hispanic women were more likely to breastfeed<br>• African–American women were more knowledgeable about SIDS<br>• although African–American and Hispanic parents had similar concerns, behaviours differed. This may help to explain the racial/ethnic disparity seen in sleep-related infant deaths.
Miladinia et al (2015)[83]	Iran	February 2011 - February 2013	To find the SIDS risk factors among families during their childcare in Iran	mothers of infants < 12 months of age	n=878	observational cross-sectional study. Convenience sampling of mothers who attended government health clinics. Data was collected by face-to-face interview using a self-structured questionnaire. Chi-square, linear regression, and ANOVA were used for analysis.	• 33.15% of parents were under 20 years of age<br>• co-sleeping practiced by 92.03%<br>• bed-sharing practiced by 49.31%<br>• side lying position used by 47.49%<br>• non-exclusive breastfeeding 45.5%<br>• significant relationship was found between bed-sharing and head covering (P<0.05); bed-sharing and prone position (P<0.05); bed-sharing and breastfeeding (under 4 months) (P<0.05); mothers' age and co-sleeping (P<0.05) mothers' age and prone position (P<0.05).<br>• Approximately 40% of infants were exposed to smoking
Möllborg et al (2011)[84]	Sweden	2003 (exact period not reported)	To examine bed-sharing at 6 months of age and the factors that are associated with bed-sharing.	Parents of infants at 6 months of age	n=5605	Radom sample of the total birth cohort in 2003. Questionnaire administered via post when child was 6 months of age. chi-square test and binary logistic regression.	• 19.8% bed-shared.<br>• correlation between breast-feeding and bed-sharing<br>• association with 3+ nightly awakenings at 6 months and bed sharing<br>• more common to share a bed if the parent was single<br>• less common to bed share if the infant was bottle-fed in the first week<br>• Never using a pacifier was associated with a higher frequency of bed-sharing.<br>• 12.4% slept alone in a separate bedroom<br>• two-thirds sleep in a separate bed in the parents' bedroom
Moon et al (2010)[85]	US; Washington, DC and Maryland	Jul2006 – Dec2008	To investigate, by using qualitative methods, perceptions about SIDS in African American parents and how these influence decisions	African American mothers with infants 0-6 months	N=83 [participated in 13 focus groups and 10 individual interviews]	Cross-sectional mixed methods study quantitative survey (mean infant age 2.6 months, range 0.5-6.0months) followed by qualitative interviews (mean age 2.6months, range 1.1-9.3months) via 13 focus groups and 10 individual, in-depth, semi-structured interviews	▪ 62.6% supine night prior; 16.9% prone<br>▪ 62.3% used a dummy<br>▪ 8.4% mothers smoked<br>▪ 33.7% never breastfed; 28.9% started but stopped; 19.3% partially breastfed at time of survey; 18.1% exclusively breastfed at time of survey<br>▪ 9.6% infants slept in a room alone night prior to survey<br>▪ 15.7% bedshared part of night prior; 14.4% bedshared entire night prior
Moon et al (2019)[86]	US; Washington DC	January 2015- December 2016	To describe the structure of networks in a cohort of mothers and to analyse associations of social network characteristics and norms with infant sleep practices.	mothers with infants <6 months of age	n= 402	hospital-based and chain-referral recruitment strategies. Mothers completed a survey about their personal social networks and infant care practices. Latent class analysis (LCA) identified unobserved network	• identified 2 a priori unknown social network types: "exclusive" (restricted) and "expansive."<br>• Mothers who were black, younger, unmarried, less educated, and of lower socioeconomic status were more likely to have exclusive networks<br>• Mothers with exclusive networks were more likely to be exposed to the norm of soft bedding<br>• Exposure to norms of non-supine infant placement, bedsharing, and soft bedding use within one's network was associated with engaging in these practices<br>• Network norms were strongly associated with infant sleep practices

Citation	Country	Study period	Study aim	Sample population	Sample size	Design/method	Key findings
						types. Binary statistics and path analysis were performed	
Moon and Omron (2002)[87]	US; Columbia	Summer 1999	To determine the prevalence and determinants of prone sleeping among infants in the District of Columbia and to ascertain what information is being provided to parents by health care professionals	parents of infants 0-6 months of age	n=126	Cross-sectional survey via survey completion when presenting for well-childcare	▪ 34.1% supine, 50.8% side, 15.1% prone to sleep the prior night<br>▪ 35.7% placed prone to sleep at least once in the past<br>▪ 48% bedshare with their mother on an adult bed<br>▪95.2% up to date with immunisations
Nelson et al (2001a)[88]	International Study; from 17 countries at 21 centres	coldest 2 months of the year from late 1995 – mid 1997	To present collected data on the key SIDS risk factors of infant sleep position and parental smoking	mothers with infants 3 months of age during the coldest 2 months of the year	n=4656 (approx. 250 families from each centre)	Cross-sectional survey with two stages – birth questionnaire and home postal questionnaire when baby was 12 weeks old	▪ Sleeping position 'last night' – 52.5% supine (range 14-89%), 37.8% side, 13% front, 3.1% other or combination of positions<br>▪ 22.3% (range 0-43%) mothers smoked at birth, 21.4% (range 0-34%) at 3 months<br>▪ 45.0% (range 14-74%) fathers smoked at birth, 41.9% (range 10%-64%)
Nelson et al (2001b)[89]	International Study; from 17 countries at 21 centres	coldest 2 months of the year from late 1995 – mid 1997	To present data on infant sleeping environment with the main focus being SIDS risk factors (bedsharing and infant using a pillow) and protective factors (infant sharing a room with adult)	mothers with infants 3 months of age during the coldest 2 months of the year	n=4656 (approx. 250 families from each centre)	Cross-sectional survey with two stages – birth questionnaire and home postal questionnaire when baby was 12 weeks old	▪ Infants room sharing with parents ranged 46% New Zealand – 98% China<br>▪ Infants bedsharing range 2% Turkey – 88% China<br>▪ Pillows least popular in Hungary 0%, Scotland 4%, Canada 8%, New Zealand 9% with most popular in Chinese samples Beijing 95%, Chongquing 95%, Hong Kong 80%<br>▪ Different types of pillows were used eg. infant vs adult pillows
Nelson et al (2005)[90]	International Study; from 17 countries at 21 centres	coldest 2 months of the year from late 1995 – mid 1997	To describe infant-feeding practices and pacifier use in varied populations and to assess factors associated with breastfeeding	mothers with infants 3 months of age during the coldest 2 months of the year	n=4656 (approx. 250 families from each centre)	Cross-sectional survey with two stages – birth questionnaire and home postal questionnaire when baby was 12 weeks old	▪ Exclusive breastmilk feeding only at approximately 3-months old 43% (range 4-80%), 20% mixed feeding, 36% formula milk fed<br>▪ Dummy use 12.5-71%; 5.1% used a dummy most of the time, 44% some of the time, 51% not at all
Norton and Grellner (2011)[91]	US; Missouri	Mar 2002 – Feb2008	To determine the prevalence of infant bed-sharing and its associations in a clinical practice	infants who presented to a family practice residency program	n=2,405	Chart reviews of de-identified electronic medical records of all infants who attended a family practice centre for any of their initial for well child visits	▪19% infant's bed-shared at 1 month; 18% at 2-3months; 12% at 4-5 months and 11% at 6-8 months. 28% documented ever bed-sharing<br>▪Only 43% documented ever breastfeeding
Nongkynrih et al (2017)[92]	India; Mangalore	Not reported	To undertake a study on knowledge and practice on maternal bed sharing among mothers whose babies are within the age group 0-3 ½ months of age	Mothers of infants <3.5 months old	n=100	Quantitative research approach with non-experimental descriptive correlative research design. Cross sectional survey.	• 89 %) of their babies always sleeps beside the mother on the same bed<br>• 100 % of their babies had slept beside the mother on the same bed at one point<br>• 50% had an average knowledge of maternal bed-sharing risks. 46% had a poor knowledge.<br>• 71% of the mothers kept their baby on his/her back while sleeping<br>• 95% of the babies sleep in a very soft mattress<br>• 66% kept soft dolls and pillows near to the baby while sleeping<br>• There is a moderate positive correlation between knowledge and practice on maternal bed sharing

Citation	Country	Study period	Study aim	Sample population	Sample size	Design/method	Key findings
Oden et al (2012)[93]	US; Washington, DC [at a hospital-based paediatric primary care site for well child visits]	Data collection dates not reported	To assess knowledge, attitudes, and practice regarding swaddling among adult caregivers of 0- to 3-month-old infants. To determine how common swaddling is practiced and to characterise the differences between those who routinely swaddle and those who do not routinely use swaddling	adult caregivers with infants aged 0-3 months old<br><br>Mean infant age 4.9 weeks old	n=103	Cross-sectional descriptive survey	▪100% had swaddled their infant at least once<br>▪83.5% use swaddling; of these 72.8% swaddle at least once daily; 44% more than twice a day<br>▪Of swaddled infants, 88.2% were placed supine; 11.8% side<br>▪ Infants not swaddled 81.6% were supine; 9.7% side; 8.7% prone
Osberg et al (2021)[94]	Norway; Oslo	May - December; 2018	To determine infants' sleeping practices in Norway, and to what extent they were exposed to potentially hazardous sleeping arrangements	Parents with infants up to 12 months of age	n=4150	cross-sectional survey. online survey. survey was marketed to the parents of infants via public health centres, websites and social media. survey consisted of 36 questions	• 2.6% routinely placed their child to sleep in a prone position.<br>• 33.2% routinely used a side position.<br>• 62.7% routinely bed shared.<br>• 14.1% were smokers at the time of the survey.<br>• 77.2% answered that bed-sharing was linked to breastfeeding.<br>• single parents, infant being older than six months and nocturnal breastfeeding increased likelihood of bedsharing.
Panaretto et al (2002)[95]	Australia; Townsville, Queensland	3 week period in Oct 1999	To assess the prevalence of SIDS risk factors in the Indigenous and non-Indigenous community of Townsville, a large remote urban centre in north Queensland, Australia	women with an infant at two shopping precincts	N=60	Cross-sectional study via single interview	▪ 0% infants <3months were regularly placed prone; Of the 33 infants between 3-12 months 44% Indigenous infants & 18% non-Indigenous infants were regularly placed prone.<br>▪ Infant sleeps in same room as parents - 93% Indigenous; 53% non-Indigenous<br>▪ Infant regularly bedshares - 77% Indigenous; 13% non-Indigenous<br>▪ Mother smoked during pregnancy – 53% Indigenous; 23% non-Indigenous<br>▪ Mother current smoker at time of interview – 60% Indigenous; 20% non-Indigenous<br>▪ Partner smokes – 41% (9/22) Indigenous; 25% (7/28) non-Indigenous<br>▪ Smoking in the home – 40% Indigenous; 20% non-Indigenous<br>▪ Breastfeeding at any time – 83% in both groups<br>▪ Exclusively or on most occasions breastfeeding at time of interview – 30% Indigenous; 47% non-Indigenous; For the 27 infants <6months at time of interview – 27% (4/15) Indigenous infants exclusively breastfeed & 33% (5/15) partially breastfed; 42% (5/12) & 25% (3/12) respectively for non-Indigenous infants
Paterson et al (2002)[96]	New Zealand; South Auckland [Middlemore Hospital]	15 Mar- 17 Dec 2000	To describe infant bed-sharing among Pacific families in New Zealand	mothers with infants 6-weeks old	n=1376	Longitudinal study via one hour in home interviews	▪ 54.9% infants shared a mattress with others<br>▪ Of infants sharing a bed – 44.6% mothers smoked yesterday; 58.9% fed breastmilk only<br>▪ Of infants not bedsharing – 55.4% mothers smoked yesterday
Pease et al (2017)[97]	UK; Bristol	February - November, 2014	To understand mothers views and decision-making process on the infant sleep environment and safe sleep messages	Mothers of infants in deprived areas of Bristol	n=20	purposive sampling using results from a brief face-to-face questionnaire with mothers at health visitor led baby clinics. Interviews were conducted by one female researcher at mothers' own homes.	▪ 75% of mothers were under 26 years of age.<br>▪ 80% of mothers were smoking at the time of the survey.<br>▪ 55% had three or more children.<br>▪ First theme: previous experience.<br>▪ Second theme: credibility of advice.<br>▪ Third theme: beliefs about disrupted routines.<br>▪ Fourth theme: beliefs about alternative strategies for reducing the risk.
Pease et al (2018)[98]	UK; Bristol	2014	To investigate mothers' knowledge of reducing the risks for sudden infant	Mothers of infants in	n=400	cross-sectional survey was carried out in deprived areas of Bristol,	• 12% mothers surveyed were classified as higher risk.<br>• Mothers in the higher risk group were less likely to breast feed, less likely to be able to cite two or more unprompted correct SIDS risk reduction strategies

Citation	Country	Study period	Study aim	Sample population	Sample size	Design/method	Key findings
			death syndrome (SIDS) and attitudes towards safer sleep practices	deprived areas of Bristol		UK. Recruitment took place in 2014 at local health visitor-led baby clinics.	• 52% of all mothers surveyed from these deprived areas in Bristol identified infant sleep position as a risk reduction strategy for SIDS<br>• 65.8% were formula fed<br>• 26.2% were exclusively breastfed<br>•52.3% said they were worried about SIDS<br>• Most mothers recalled a conversation about safer sleep in this pregnancy (93.3%) with either a health visitor (62.5%) or midwife (67.3%).<br>• There was little difference between the two groups regarding being worried about SIDS, recalling a conversation about safer sleep with a health professional<br>• 6.3% did not name any correct strategies for SIDS risk reduction, 10.5% named one correctly, 29% named two correctly and 54.3% could name three or more correct strategies.<br>• Mothers in the higher risk group were more likely to agree that tummy sleeping was okay
Phares et al (2004)[99]	US; 8 states - Alabama, Colorado, Florida, Hawaii, Illinois, Maine, Nebraska and North Carolina	2000-2001	To identity the nature and extent of disparities in maternal behaviours that affect maternal or infant health	mothers of infants 2-3 of age	n=40,356	Mixed mode data collection via posted self-administered surveys with follow-up telephone interview for non-responders	▪ Smoked during last 3 months of pregnancy 9.0% - 17.4%<br>▪ Drank any alcohol during last 3 months of pregnancy 3.4% -9.9%<br>▪ Breastfeeding initiation 54.8% - 89.6%<br>▪ Supine position 49.7% -74.8%<br><br>While prevalence data cannot be used to identify causes or interventions to improve health outcomes they can indicate the magnitude of disparities and population groups that should be targeted for intervention. Results revealed marked socioeconomic, racial, ethnic and age disparities within the four critical maternal health-related behaviours. Further research is needed to examine factors experienced by women that prevent or enhance health-promoting behaviours.
Pretorius et al (2020)[100]	N/A	May-19	To describe and analyse conversations among mothers engaged in discussions about SIDS on a Facebook mother's group.	Mothers of infants who were members of a Facebook Mothers group	n=512	extracted and analysed 20 posts and 912 comments from 512 mothers who participated in a specific Facebook mother's group and engaged in conversations about SIDS. 2 reviewers who coded the data using qualitative descriptive content analysis.	• The theme of social support emerged, specifically informational and emotional support.<br>• There was widespread discussion regarding infant sleep products and monitoring devices.<br>• Embedded within conversations were (1) confusion among commonly used medical terminology, (2) the practice of unsafe infant sleep, (3) inconsistency in provider communication about SIDS, and (4) maternal anxiety regarding SIDS.<br>• Mothers openly asked for recommendations or feedback regarding infant sleep practices, use of baby products or monitoring devices, or other topics related to SIDS or safe sleep.<br>• Mothers shared information provided by their health care provider regarding SIDS, safe sleep, and the use of baby products.<br>• Mothers also shared information by providing their own personal definitions or beliefs regarding SIDS or safe sleep
Provini et al (2017)[101]	US, Caribbean, Mexico, Central and South America	January 2011 - March 2014	To assess the association between maternal birth country and adherence to the American Academy of Pediatrics (AAP) safe sleep recommendations in a national sample of Hispanic mothers	Hispanic mothers of infants residing in the US	n=907	stratified, 2-stage, clustered design. surveys (administered 2–6 months postpartum) were received from mothers who self-identified as Hispanic/ Latina. prevalence estimates and aORs were determined for infant sleep position, location, breastfeeding, and maternal smoking.	• When compared with U.S.-born mothers, mothers born in the Caribbean and Central/South America were significantly more likely to room share without bed sharing<br>• Caribbean-born mothers were significantly less likely to place infants to sleep supine<br>• Mothers born in Mexico and Central/South America were significantly more likely to exclusively breastfeed<br>• Caribbean-born mothers were significantly less likely to breastfeed<br>• majority of Hispanic mothers reported infant care practices consistent with AAP recommendations for both sleep position (supine, 73.8%) and location (room sharing without bed sharing, 70.0%).<br>• One- quarter (25.3%) of the Hispanic mothers reported bed sharing.<br>• 6.4% of Hispanic mothers reported smoking during pregnancy.

Citation	Country	Study period	Study aim	Sample population	Sample size	Design/method	Key findings
Raines (2018)[102]	US	January-April, 2016	To explore factors that influence parental behaviours related to newborn sleep positions and environments in the home after hospital discharge	Mothers of infants	n=60	descriptive qualitative study conducted by telephone. Participants recruited from the patient population of the mother–baby unit	• 87% participants mentioned grandmothers as influences.<br>• Thirty-two of the participants indicated that grandmothers influenced their decisions to position their infants in a prone or side-lying position to prevent choking.<br>• father or husband was a recurrent influence mentioned by 80% participants.<br>• The findings indicate that although new parents may know about safe sleep recommendations, their actions and behaviours in the home are not consistently guided by that knowledge but rather by other influential factors.
Roberts and Upton (2000)[103]	UK; at a single large maternity unit, located on the fringe of 3 large urban areas	Dates of data collection not reported - (during a 12 week period)	To examine the level of awareness mothers have of the factors that relate to the prevention of SIDS and how women are currently caring for their infants.	mothers who recently delivered an infant	n=206	Cross-sectional study via community conducted interviews by midwives when doing home visits at the 28-day evaluation and discharge visit	▪ 75.7% supine, 0.5% prone, 9.7% side, 14.1 % combination side/back<br>▪ 29% reported currently smoking<br>▪ 33% reported there was a smoker other than mum living in the household<br><br>Knowledge of the preventative factors was generally high; a quarter of mothers were not adopting the recommended sleep position and a third continued to smoke.<br>The level of advice provided by healthcare professions was perceived to be low with instances of inappropriate professional practice possibly perpetuating incorrect sleep position.
Robida and Moon (2012)[104]	US; Washington DC, and Maryland.	Between April 2006 and May 2010	To investigate factors influencing African-American parents' knowledge, attitudes and practice regarding infant sleep position and determine if these differ by socioeconomic status (SES).	African-American parents of infants (0-6 months).	n=412	A cross-sectional sample. Individual surveys of knowledge, attitudes and practice. Significance tests performed using STATA/SE software.	• There is no difference in infant sleep position practice by socio-economic status in African-Americans.<br>• Sleep position practices in African-American families do not differ by SES.<br>• 62.9% supine sleeping.<br>• 8.5% side.<br>• 9.5% prone.<br>• Higher SES participants have better knowledge of SIDS.
Rohana et al (2018)[105]	Malaysia; Kula Lumpur	December 2016 - August 2017	To assess the knowledge and practises regarding SIDS risk reduction measures among parents of preterm infants discharged from the neonatal intensive care unit (NICU) of University Kebangsaan Malaysia Medical Centre (UKMMC), Kuala Lumpur, Malaysia	Parents of preterm infants.	n=80	Cross-sectional. Self-administered survey questionnaire to assess their knowledge and practises regarding SIDS risk reduction.	▪ 61.3% of parents had heard of SIDS prior to the study<br>▪ Information about SIDS was gathered mostly from social media (67.3%)<br>▪The only recommended practice that few parents followed was regular use of a pacifier (19%).<br>▪ Only 47.5% of parents put children to sleep in their own bed<br>▪Only 44% of parents were categorised as having 'good knowledge' of SIDS and safe sleeping (>5 answers correct out of 9)<br>▪21.3% put child to sleep in prone position<br>▪21.3% put child to sleep in prone position
Ruiz Botia et al (2020)[106]	Spain	February 2017 - February 2018 and June - July 2018.	To identify the adherence of families to current sleep recommendations in a sample of infants.	Parents of infants under 12 months residing in Spain.	n=640	questionnaires were self-administered and available online. participants recruited via clinics responsible for delivering the well-child programme at the Hospital Universitario General de Catalunya, childcare centres, as well as social networks.	▪48.3% of infants aged less than 6 months were exclusively breastfed<br>▪18.0% received other foods in addition to BF<br>▪33.7%were no longer breastfed at all<br>▪ In the group aged 6 -11 months, 52.6% were breastfed ▪only 10.4% of families of infants aged 0-5 months and 3.1% of families of infants aged 6-11 months followed all the recommendations for SIDS<br>▪ 48.6% laid babies to sleep supine<br>▪ 29.8% shared a sleep surface with their infants.<br>▪ 53.4% used a pacifier<br>▪13.3% of mothers were smokers

Citation	Country	Study period	Study aim	Sample population	Sample size	Design/method	Key findings
Salm Ward and Ngui (2015)[107]	US; Wisconsin	2007 to 2010	To examine racial differences in bed-sharing prevalence and characteristics from data collected after the AAP's explicit 2005 recommendations against bedsharing using the Wisconsin PRAMS.	mothers of a live infant who was delivered in hospital in the previous 2-4 months	n = 2,486 (n=806 non-Hispanic African American n=1,608 non-Hispanic Whites)	Retrospective analysis of PRAMS data from Wisconsin. Cross sectional study. Chi-squared tests to determine correlation. Mixed-mode data collection method via mailed questionnaires and telephone follow-up for non-respondents	• Significantly more African Americans (70.6 %) reported bed-sharing compared to Whites (53.4 %)<br>• Bed-sharing Whites were also more likely to place their infant's non-supine to sleep than non-bed-sharing Whites<br>▪ 88.7% African American supine position, 92.4% Whites<br>• For African Americans, partner and maternal education of 13–15 years was associated with increased odds of bed-sharing.<br>▪ 21.7% African American breastfeed, 51.7% Whites<br>• For Whites, partner stress, breastfeeding, income of $35,000–$49,999, being unmarried, needing money for food, and non-supine sleep were associated with increased odds of bed-sharing
Salm Ward et al (2018)[108]	US; Georgia	2004 to 2013	To describe trends and factors associated with breastfeeding (initiation and exclusivity) and infant sleep practices (sleep position and bed-sharing) in the state of Georgia.	mothers of infants (<12 months) in Georgia.	n=4643	Retrospective analysis of PRAMS data from the Georgia Department of Public Health. Cross sectional study. Chi-squared tests to determine correlation.	• From 2004 to 2013, there was a slight decrease in breast-feeding (5.7% decrease)<br>• From 2004 to 2013, there was a slight decrease in the pro-portion of mothers who reported that they most often put their infant to sleep on their back (from 59.7% in 2004 to 48.9% in 2013)<br>• The proportion of mothers who reported never bed-sharing increased significantly from 26.7 to 45.1% over the period
Salm Ward et al (2016)[109]	US; Georgia	2004 to 2011	To examine the prevalence and characteristics of bed-sharing among non-Hispanic Black and White infants in Georgia, and the differences in bed-sharing and sleep position behaviours prior to and after the AAP 2005 recommendations against bed-sharing.	mothers of infants (<12 months) in Georgia.	n=6595	Retrospective analysis of PRAMS data from the Georgia Department of Public Health. Chi-squared tests to determine correlation.	• Significantly more Black mothers(81.9 %) reported ''ever'' bed-sharing compared to White mothers (58.9 %),<br>• Bed-sharing rates declined significantly between 2000–2004and 2006–2011 for Blacks and Whites after AAP Recommendations<br>• Bed-sharing predicting factors for Blacks included infant age, pregnancy intention, number of dependents, and use of Women, Infant and Children (WIC) Services.<br>• Bed-sharing predicting factors for Whites included infant age, maternal age, financial stress, partner-related stress, and WIC.
Sawaguchi et al (2002)[110]	Japan; Yokohama	Oct 1996 – Apr 1997 first phase; 1998-1999 second phase	To observe the trends in risk factors for SIDS that may exist in the child rearing environment in Japan	mothers with infants 3 months of age during the coldest 2 months of the year	n=96 first phase n=49 second phase	Cross-sectional survey via home postal questionnaire when baby was 12-weeks-old. Second phase completed after a SIDS prevention campaign	▪ In 1996 - 4.2% prone, 10.5% side, 85.3% supine; 1998 – 2.0% prone, 2.0% side, 95.9% supine<br>▪ Smoking – 9.4% mothers, 47.9% fathers smoked 1996; 0% mothers, 51.0% fathers smoked 1998<br>▪ Exclusively breastfed 53.1% 1996 & 67.3% 1998; Bottle fed only 10.4% 1996 & 8.2% 1998<br>▪ Room sharing with parents – 89.0% 1996, 100% 1998<br>▪ Bedsharing with parents – 46.0% 1996, 60% 1998<br>▪ Uses a pillow – 59.0% 1996, 58.0% 1998<br>▪ Uses a dummy – 8.0% 1996,
Schluter et al (2007)[111]	New Zealand; South Auckland [at Middlemore Hospital, a larger tertiary hospital]	15Mar – 17Dec 2000	To report infant care practice prevalence for known modifiable SIDS risk factors among a generally disadvantaged yet low-SIDS rate population of mothers with Pacific infants	mothers with infants approximately 6 weeks post-partum	n=1,376	Cross-sectional design via maternal self-report 1 hour in home interviews	▪ Maternal smoking 29%<br>▪ Breastfeed only 50%; Formula only 13%; Breastmilk and formula 38%<br>▪ Supine 36%, side 47%, prone 1%, 16% no special way<br>▪ Sleeps alone in own bedroom 2%; in parents' bedroom 94%; Lounge room 2%; Other 2%<br>▪ 55% bed-shares- 12% with a smoking mother, 38% non-smoking mother, 5% with others; 45% sleep alone<br>▪ Bed type – 11% bassinet, 1% carrycot, 28% cot, 7% baby's bed, 45% parent's bed, 3% baby's mattress on parents bed<br>▪ uses a dummy 27%<br>▪ uses a sheepskin 5%

Citation	Country	Study period	Study aim	Sample population	Sample size	Design/method	Key findings
Schluter and Young (2002)[112]	Australia; Queensland	Oct 2001	To pilot a tool intended for a state-wide prevalence study of infant care practices associated with SIDS and to estimate the prevalence of infanct care practices that are known to be associated with SIDS for a group of Queensland primary caregivers. The results will be used to determine whether the RTR messages have been adopted by these caregivers	primary caregivers with infants aged 1-6 months attending 1 of 3 metropolitan community immunisation clinics	n=36	Cross-sectional survey design via paper-based questionnaire	▪71% infants usually placed supine; 17% prone & 11% side<br>▪14% mothers smoked during pregnancy & 11% were current smokers<br>▪71% infants were placed with their feet to the foot of the cot<br>▪57% of infants slept in their own room over the last 2 weeks<br>▪57% used a dummy in the last 2 weeks<br>▪91% of infants did not use a pillow last night<br>▪11% of infants slept with soft toy/s last night<br>▪89% infants were 8-12 weeks at their first immunisation visit<br>▪86% infants exclusively BF at hospital discharge; 69% at 4 weeks 25% over last 2 days<br>▪9% of infants main night-time bed over the last 2 weeks was the parent's bed<br><br>Deficiencies in some primary caregivers' knowledge and practices relating to infant care practices associated with reduced SIDS risk were identified and may directly contribute to Qld's relatively high SIDS rate.
Shapiro-Mendoza et al (2015)[113]	US	1993-2010	To investigate the US prevalence of and trends in bedding use.	Primary caretakers of infants in the US	n=18952	used data from the NISP (National Infant Sleep Position) Study - annual, cross-sectional telephone survey conducted from 1993 to 2010. Random sample of households chosen from a purchased list containing public information from birth records, infant photography companies, and formula companies.	• percentage of infants covered with thick blankets or quilts/comforters declined significantly from 1993 through 2010 (from 85.9% to 54.7%).<br>• 37.6% most frequently reported types of bedding covers were thick blankets<br>• 74.2% slept in a crib or bassinet<br>• 72.6% were placed to sleep supine<br>• 86.5% did not share a sleep surface (ie, they slept alone)
Shields et al (2005)[114]	US; Kentucky [recruited from a metropolitan hospital]	14Oct – 10Nov 2002	To ascertain the prevalence of infant care practices in a metropolitan community in the US with attention to feeding routines and modifiable risk factors associated with SUID (specifically, prone sleeping position, bed sharing, and maternal smoking)	women of infants who were placed in the well-infant nursery	n=189 recruited n=185 at 1-month (93.9%) n=147 at 6-months (75.1%)	Telephone survey at 1-month and 6-months postpartum; those unable to be contacted by phone were sent written surveys	▪ 50.8% bedshared at 1-month; 17.7% at 6-months<br>▪ 67.0% supine, 14.0% side, 0% prone, 18.9% combination at 1-month; 68.0% supine, 8.8% side, 11.6% prone, 11.6% combination<br>▪ Bed type – crib/bassinet 47.0% (80.9%), parents bed only 10.3% (10.2%), combination 38.9% (8.2%), other 3.8% (0%) at 1-month (at 6-months)<br>▪Sleeping room – parents' room 71.3% (46.3%), infants own room 15.7% (53.7%), living room 2.2% (0%), unknown 10.8% (0%) at 1-month (at 6-months)<br>▪26.5% reported smoking at 1-month, of these 14.3% smoked in the house; 23.8% smoked at 6-months, 14.3% smoked in the house<br>▪ 73.0% formula fed only at 1-month; 13.5% exclusively breastfed; 0% exclusively breastfed at 6 months – 4.1% fed breast/semisolids, 87.8% formula/semisolids, 7.5% combinations; 57.3% fed in bed at 1-month, 34.7% at 6-months
Sivan et al (2004)[115]	Israel	Sep2001 – Dec2001	To investigate the role of religious way of life on parents' adherence to the recommendations to put their baby to sleep in a non-prone position and to evaluate the significance of other contributing factors in relation to the level of religiousness in the Jewish population	parents of 2-month-old infants	n=608	Longitudinal survey by completion of questionnaire via telephone interviews	▪80.4% non-religious or traditional infants were placed in a non-prone position at 2 months; 71.4% at 4 months; 68.3% at 6 months<br>▪68.8% of orthodox infants were placed non-prone at 2 months; 64.6% at 4 months; 62.7% at 6 months<br>▪56.3% of ultra-orthodox infants were placed non-prone at 2 months; 50.0% at 4 months; 39.8% at 6 months

Citation	Country	Study period	Study aim	Sample population	Sample size	Design/method	Key findings
Smith et al (2012)[116]	US; South Carolina	1996-2007	To examine racial differences in trends and predictors of prone and lateral infant sleep positioning among South Carolina mothers and infants	Caregivers of infants under the age of 12 months in South Carolina	n= 14,648	Data came from the 1996–2007 South Carolina Pregnancy Risk Assessment Monitoring System (PRAMS) survey (cross-sectional). Linear trends in sleep positioning were assessed using separate logistic regression models for each race.	• The prevalence of prone positioning among whites declined from 28.5% in 1996 to 19.5% in 2007, and the prevalence of lateral positioning dropped from 43.3% in 1996 to 13.8% in 2007<br>• The prevalence of lateral positioning decreased among black infants, dropping from a high of 50.5% in 1997 to 20.0% in 2007<br>• In 1996 less than one-third (28.2%) of white infants were put to sleep in the supine position. By 2007, the prevalence of supine positioning had increased to 66.7%<br>• The prevalence of supine positioning increased among black infants over the study period from 22.6% in 1996 to 47.1% in 2007.<br>• Predictors of white infant sleeping position included: mother's age, marital status, household poverty index, rural/urban status of residence county, smoking after pregnancy, initiation of prenatal care in the first trimester, year of infant birth, infant's gender, and infant's age<br>• Predictors of black infant sleep position included: mother's age, household poverty index, rural/urban status of residence county, and infant's age
Smith et al (2016)[117]	US; 32 US birth hospitals	Jan2011 – Mar2014	To describe the prevalence of breastfeeding and sleep location practices among US mothers and the factors associated with these behaviours, including advice received regarding these practices	mothers with infants 2-4 months old	n=3,218	Stratified clustered design to obtain a nationally representative sample of mothers of infants, while over sampling Hispanic and non-Hispanic black mothers survey completed either online or by telephone once their infant was at least 60 days old	▪ 30.5% exclusively breastmilk, 29.5% combination of formula and breastmilk; 40.0% formula only<br>▪ 65.5% room share but sleep in own bed, 20.7% bedshare whole or part night, 13.7% sleep in own room,<br>▪All sleep surfaces used in past 2 weeks – 55.2% crib, 39.1% adult bed/mattress, 38.1% bassinet, 36.1% car seat, 33.2% pack and play, 10.2% sofa, 8.7% cradle, 8.0% co-sleeper
Smylie et al (2014)[118]	Canada	2006–2007	To examine the association between maternal education and non-supine infant sleep position, and examine patterns of effect modification with additional sociodemographic, maternal, infant, and health services predictors	First time Canadian mothers of infants aged 5-9 months	n=6334	Used data from the Canadian Maternity Experience survey. Women selected at random, survey administered via telephone. Using logistic regression, we developed multivariate models for 3 maternal education strata.	• Level of maternal education was significantly and inversely related to non-supine infant sleep position<br>• Overall, 22.6% of women reported putting their infant in a non-supine sleep position during the first 4 months<br>• Women with less than a high school education were more than twice as likely to put their baby in a non-supine sleep position than women who had completed postsecondary education<br>• For the less-than-high-school-education stratum, significant predictive factors of non-supine sleep position included not being married, living rural, late initiation of prenatal care<br>• For the stratum of women with completed high school education, significant predictive factors of non-supine sleep position included multiparity, stressful life events, living with a smoker during pregnancy, and no contact from a health care provider after birth
Specker et al (2020)[119]	US; South Dakota	Throughout 2017	To determine the prevalence of safe sleep practices among South Dakota mothers, and the impact that education from their healthcare provider had on infant safe sleep practices as defined by the American Academy of Pediatrics (AAP).	Mothers of infants under 12 months born in South Dakota	n=1,131	Used PRAMS data from South Dakota 2017. Data were weighted to obtain state-wide and race-specific (white, non-Hispanic; American Indian; other races) prevalence rates.	• 87.6% of South Dakota mothers placed their infant on their back to sleep.<br>• Mothers of other races were less likely to place their infant to sleep on their back compared to white mothers and American Indian mothers<br>• 62.4% of mothers stated that their infant always slept alone<br>• 76.5% of mothers room shared with their infant<br>• 47.7% of infants were put to sleep without blankets, toys, cushions, pillows, or crib bumper pads<br>• 89.4% of mothers breastfed or pumped breast milk even for a short time<br>• In total, 82.1% of mothers did not smoke the last three months of pregnancy or after pregnancy and their infants were not exposed to environmental cigarette smoke<br>• 85.7% of mothers attended 80 percent or more of their prenatal visits<br>• 7.7 percent of South Dakota mothers giving birth in 2017 met all eight of the AAP

Citation	Country	Study period	Study aim	Sample population	Sample size	Design/method	Key findings
							recommendations regarding safe sleep and 18.9 percent met four or fewer of the eight recommendations.
Sperhake et al (2009)[120]	Germany; Hamburg	1996, 1998, 2001 & 2006	To monitor data on risk-related behaviour in the population of Hamburg, Germany, in order to respond to changes quickly and to estimate the effectiveness of prevention activities, allowing an estimate of the prevalence of risk factors in an urban population, both transversally and vertically	parents of infants <1 year attending routine medical check-ups	n=7484	Participating practices were provided with standardised, single-sheet questionnaires concerning risk-related behaviour which were filled in by the staff of the practice by interviewing parents	▪ The prevalence of infants sleeping prone declined from 8.1% in 1996 to 3.5% in 2006, indicating local campaigns have proved effective.<br>▪ The supine position became the most freq. chosen sleeping position with 34.0% in 1996 increasing to 76.5% in 2006<br>▪ Most parents who still chose the prone sleeping position for their infants were aware of it being a specific risk for SIDS (81.7% in 2006), Only 0.6% of caregivers chose the prone sleeping position and were not aware of its risk (3.4% in 1998)
Strömberg Celind et al (2017)[121]	Sweden	April 2012 - October 2014	To examine to what extent the recommendations were followed by parents and to see if there was room for improvement.	Primary caretakers of infants <12 months	n=710	Cross-sectional sample. Participants recruited from 28 child healthcare centres around Sweden. Participants filled in a paper questionnaire. Statistical analysis using chi-squared tests.	• in the first three months, 1.3% of the infants were placed in the prone sleeping position and 14.3% were placed on their side. By three to five months, this had risen to 5.6% and 23.6%<br>• In the first three months, 83.1%were breastfed, 84.1% used a pacifier and 44.2% shared their parents' bed, while 5.8%slept in another room.<br>• Bed sharing was more likely if infants were breastfed and less likely if they used pacifier<br>• s. During pregnancy, 2.8% of the mothers smoked and the mothers who had smoked during pregnancy were less likely to bed share.
Tipene-Leach et al (2010)[122]	New Zealand; Manukau region	21 July -31 Dec 2008	To determine what Māori mothers know about SIDS prevention and to determine their SIDS-related child care practices	mothers of infants either 6-8 weeks of 3-4 months of age	n=299	Cross-sectional survey via telephone interview and home visit if phone contact was not made	▪ Overall, 13% 'usually' slept their infants in some combination of sleep position that included front<br>▪27% of mothers who cited back sleeping as preventive for SIDS did not sleep baby on the back<br>▪ 53% mothers smoked in pregnancy; 51% smoked in last 24 hours<br>▪91% mother reported ever BF their infant, only 51% of 3–4-month-old infants were still BF at time of survey<br>▪ 65% mothers indicated some bed sharing last night, with 39% of those sharing for >2hrs
Tirosh et al (2000)[123]	Israel; at five well baby clinics representing four different socioeconomic districts	Sep 1996-Feb 1997	To compare the prevalence of three risk factors, ie. positioning, night dressing/ covering and heating practices in these ethnic groups	parents of infants 1 week – 4 months of age	n=408	Cross-sectional survey via questionnaire when parents attend a well-baby clinic	▪ Significantly more Jewish infants shared a room with siblings (31.8%) compared to Arab infants (11.1%<br>▪ 58.4% Arab infants slept in parents' room compared to 37.4% Jewish infants<br>▪ Most infants of both groups slept in their own beds (98.8% Jewish infants, 97.9% Arab infants)<br>▪ 59% of cohort reported routinely putting infants to sleep in a supine position<br>▪ More Jewish mothers than Arab mothers reported the prone position preferred by the infant
Tully et al (2015)[124]	US	2010-2012	To compare maternal report of planned and practiced home sleep locations of infants born late preterm (34 0/7 to 36 6/7 gestational weeks) with those infants born term (C37 0/7 gestational weeks) over the first postpartum month.	Mothers of infants (born at term and late preterm) under six months of age.	n=56 (first interview). n=46 (follow up interview)	Sample collected from an ongoing observational study about maternal wellbeing. Open-ended semi-structured maternal interviews and questionnaires following birth and then one month later.	▪ primary reason for unplanned bed sharing was to soothe night-time infant fussiness.<br>▪ 89.3% mothers planned to room share.<br>▪ 91.1% practiced room sharing. ▪ ~12% planned to bed share.<br>▪ No difference was observed in preterm and term bedsharing practice.<br>▪ 47.7% practiced bed sharing in the first month postpartum.

Citation	Country	Study period	Study aim	Sample population	Sample size	Design/method	Key findings
van Sleuwen et al (2003)[125]	Netherlands; 8 regions with a representative sample of concentrations of Turkish and Moroccan populations [at 28 well-baby clinics]	Jun-Oct 1999	To investigate what other differences between ethnic groups exist in infants care practices	Families with infants a mean age of: Dutch 7.45, Turkish 7.42, Moroccan 5.74	n=319	Cross-sectional study design via face-to-face interviews	▪ Mother smoking at time of interview – 32.9% D, 29.6% T, 1.9% M; Mother smoking during pregnancy – 26.7% D, 22.2% T, 1.9% M<br>▪ Sleep position: D – 84.6% supine, 5.3% prone, 10.1% side; T – 74.0% supine, 6.0% prone, 20.0% prone; M – 69.4% supine, 4.1% prone, 26.5% side<br>▪ Sleeping sack 70.5% D, 52.7% T, 35.2% M<br>▪ Pillow 2.5% D, 31.5% T, 11.1% M<br>▪ Cot buffer 12.6% D, 45.7% T, 46.5% M<br>▪ Soft Mattress 5.7% D, 16.4% T, 31.5% M<br>▪ Occasional bed-sharing 40.4% D, 37.0% T, 38.9% M<br>▪ Parental room sharing – 0-2months 42.9% D, 22.2% T, 54.5% M; 2-3months 17.6% D, 25.0% T, 0% M, >3months 7.3% D, 26.8% T, 26.2% M<br>▪Dummy use – 0-3months 40% D, 76.9% T, 50% M; 3-6months 34.7% D, 58.3% T, 38.4% M<br>▪Swaddling – 6.2% D, 10.9% T, 14.8% M
Vernacchio et al (2003)[126]	US; Boston, Lowell, Lawrence, Toledo	1995-1998	To describe sleep positions among low-birth-weight infants, variations in sleep position according to birth weight, and changes in sleep position over time. To analyse risk factors and influences associated with prone sleep.	mothers who delivered live infants weighing <2500g	n=907 enrolled – n=744 at 1 month n=720 at 3 months n=689 at 6 months	Prospective, longitudinal cohort study	▪Prevalence of supine sleeping at 1 month 23.8%, 3 months 37.9% and 6 months 50.2%<br>▪Prone sleeping decreased among low-birth-weight infants from 1995-1995; however very low birth weight infants are more likely to sleep prone
Varghese et al (2015)[127]	US; Staten Island	January - October; 2013	To explore beliefs and attitude toward infant sleep safety and sudden infant death syndrome (SIDS) risk reduction behaviours among caregivers of newborns and infants.	adult caregivers, including parents and grandparents, of newborns under the age of 12 months.	n=121	Self-administered cross sectional questionnaires distributed to caregivers in the inpatient setting before a newborn's discharge from the hospital. Descriptive statistics were used to examine the responses to the questionnaire as well as demographic information, including socio-economic data	• Despite an overall favourable attitude toward safe sleep practices, a majority disagreed with use of pacifiers (53%) and believed that swaddling (62%) as well as the use of home monitors (59%) are acceptable practices.<br>• 22% of the respondents disagreed with placing an infant on their back during sleep.<br>• Respondents who recalled being taught about safe sleep believed that safe sleep behaviours are effective and had significantly more confidence in their ability to implement safe sleep behaviour<br>• Caregivers with less formal education worried more about their infant choking in the supine position<br>• caregivers with higher formal education perceived more infant vulnerability to SIDS<br>• Caregivers with annual incomes ofo$80,000 per year worried more about infant choking while in the supine position were more concerned about making the back of the baby's head flat and had less confidence in their own ability to implement safe sleep behaviours .
Vilvens et al (2020)[128]	US; Ohio	Autumn 2016	To understand why parents might not be practicing safe sleep by exploring knowledge, attitudes, behaviours and current practices of infant sleep; identifying what messages and information would motivate behaviour change; and describe preferences for setting and delivery modality of safe sleep information.	Parents/primary caretakers of babies<12months.	n=124	Cross-sectional. Participants recruited at community events/festivals. Semi-structured qualitative interview.	▪ 26% practised bed sharing<br>▪ 72% placed baby to sleep on their back<br>▪26% would prefer to receive information from a one-on-one consult with a medical professional<br>▪ 20% would prefer to receive information via the internet.<br>▪ 14% place baby to sleep with blanket/pillows in the crib
Von Kohorn et al (2010)[129]	US; Alabama, Connecticut, Michigan,	2006-2008	To determine the relationship between the advice mothers receive about infant sleep position	mothers, with infants <8 months at Women Infants	n=2299	Cross-sectional, face-to-face interviews	▪Advice for exclusively supine infant sleep position from family, doctors, nurses, or the media was associated with usually placing an infant supine to sleep.<br>▪Mothers who believe their infant is comfortable supine are more likely to place their infant on their back to sleep

Citation	Country	Study period	Study aim	Sample population	Sample size	Design/method	Key findings
	Mississippi, Texas		and the actual position they place their infants in to sleep and to understand modifiers of that relationship, especially beliefs about infant comfort and safety.	and Children Supplemental Nutrition Program centres			▪Mothers who believe their infant will choke on its back are less likely to place their infant supine.
Walcott et al (2018)[130]	US; Georgia	August - October 2016	To assess the knowledge and behaviours regarding sleep position and sleep location to identify the parent characteristics and specific hospital intervention components most closely associated with positive safe sleep knowledge and behaviour	primary caretakers of all infants born in Georgia	n=420	Web-based survey sent to all mothers who gave birth in Georgia during the study period. Cross-sectional.	▪ 95% of participants received safe sleep information from hospital.<br>▪ Positive correlation between parental age and safe sleep knowledge.<br>▪ White parents were more knowledgeable about safe sleep practices.<br>▪ High correlation between knowledge of safe sleep and practice of supine sleeping.<br>▪ Parents who received a bassinet in hospital were four times more likely to room share.<br>▪ 89% of respondents placed infant to sleep in supine position.
Wennergren et al (2021)[131]	Sweden	Throughout 2018	To study a new birth cohort so that we could identify infant care practices with regard to the position they slept in, where they slept and what bedding arrangements were used if they slept in the same bed as their parents	Parents of infants born in 2018 in Sweden.	n=3590	longitudinal and population-based cohort study of children born in western Sweden in 2018. Half of the birth cohort in Sweden was randomly selected to participate in postal questionnaires. Questions were asked about the childs sleeping habits at both three and six months of age.	▪ 60% of parents placed child to sleep in supine position at 3 months. This decreased to 40% at 6 months.<br>▪Any use of prone sleeping position was 6% at 3 months to 11% at 6 months<br>▪At 3 months of age, 54% of parents room shared.<br>▪ At 3 months, 43% bed shared. At 6 months, this decreased to 33%<br>▪Bed sharing rose 13% from 2004<br>▪ Tendency to bed shared correlation with increased tendency to breastfeed.<br>▪There was a negative association between dummy use and bed sharing .<br>▪ Of those who slept in their parents' bed, 42% slept in a baby nest and 42% in close contact with their parent, while 15% of the parents tried to create a separate space for their infant without using a baby nest
Willinger et al (2000)[132]	US; the 48 contiguous states	1994-1998	To examine sociodemographic characteristics, motivation, and message exposure to ascertain which factors influenced a caregiver's choice of infant sleep position after implementation of the campaign.	Caregivers with infants born in last 7 months	n= >1000/year	Annual nationally representative telephone surveys	▪Between 1994-1998 prone placement declined from 44% to 17% among white infants and from 53% to 32% among black infants<br>▪Reports of supine recommendations from at least 1 source doubled from 38% to 79%.<br>▪Infant comfort given as reason for prone placement by 82% of caregivers<br>▪Physician recommendation 'supine not prone' had strongest influence and associated with decreased prone placement and increased supine placement
Wilson (2000)[133]	Canada; women living on a Cree reserve	1996	To identify contemporary Cree infant care practices and any risk factors associated with SIDS	women with infants under 12 months of age	n=70	Cross-sectional survey via in-home interviews	▪ 72% initiated breastfeeding with 35% reporting breastfeeding for <3months<br>▪ 80% sleep with their mothers, frequently with other siblings<br>▪ All infants slept supine and are swaddled with a thin blanket
Woods et al (2015)[134]	US	Not reported	To use data collected for larger projects in a secondary data analysis to compare self-reported safe sleep data from a community-based social service agency setting and a primary care setting	Primary caretakers of infants in sample region	n=166 (PRAMS respondent) n=79 (primary care centre respondent)	Cross-sectional PRAMS–based survey data from a community social service agency and survey data from primary care centres were compared using descriptive statistics.	• Safe sleep position responses did not differ significantly between the community-based and primary care centre–based samples<br>• Reported bed sharing was significantly higher in the community sample<br>• Black race was determined to be significantly associated with increased unsafe sleep positioning<br>• The community centre cohort was the only significant predictor of bed sharing<br>• 85% PRAMS respondents reported they lay their baby on his or her back while sleeping<br>• 64% PRAMS respondents never shared a surface with their infant. 30% sometimes shared a surface. 6% always shared.

Citation	Country	Study period	Study aim	Sample population	Sample size	Design/method	Key findings
						Two binary logistic regressions were performed to examine the association between demographic differences and safe sleep position/bed sharing	• 33% of PRAMS respondents practised all AAP Safe Sleep recommendations<br>• 20% of primary care centre respondents where compliant with AAP Safe Sleep recommendations<br>• 30% of respondents in the community setting (compared with 5% in primary care location) reported bed sharing
Wright et al (2014)[135]	UK; an outpatient chiropractic teaching clinic	Sep 2011 & Jan 2012	To investigate the sleeping position of infants attending an outpatient clinic, considering the influences of the back to sleep campaign	parents with infants <1 year of age	n=678	Cross-sectional survey via paper-based questionnaire using a convenience sample	•50% of infants' primary sleeping position was supine; 19% prone
Yikilkan et al (2011)[136]	Turkey; Istanbul	Not reported	To determine the knowledge and attitude of parents and health professionals about SIDS and associated risk factors.	Mothers of infants and health professionals from government Mother & child clinics	n=324	This is a descriptive, cross-sectional study. A total of 174 health professionals and 150 mothers were enrolled in this study. Mothers' data were collected by telephone interview and health-care professionals were interviewed by the same investigator. Participants recruited via random selection of government funded health care centres.	• Only 39% of mothers were aware of SIDS.<br>• 46% of the mothers preferred a supine sleeping position for their infant<br>• 16% of the parents were bed-sharing with their infants.<br>• 73% of health professionals selected side, 17% supine and 10% prone sleeping position as the safest sleeping position.<br>• frequencies for awareness of risk factors were: bed-sharing (75%), soft bedding (70%), pillow use (52%), toys in bed (90%), high room temperature (67%) and smoking (88%).<br>• 82% claimed that they had never been advised on sleeping position by the health professionals.<br>• 55% of the infants were exclusively breast-fed. Frequency of exclusive breast-feeding was significantly less among pacifier users<br>• Eighty-one percent of health-care professionals rated recommendation of sleeping position as absolutely necessary, 8.6% as necessary and 10.3% as unnecessary.
Zoucha et al (2016)[137]	US	Not reported	To explore the cultural influences of safe sleep practices by African-American caregivers of children under 2 years old. Explore the role of health care professionals in promoting safe sleep	African-American primary caregivers of children under 2 years	n=19	A voluntary convenience sample. Focused ethnography. This included fieldwork, participant observation, participant and family interaction, individual and follow-up focus groups for data analysis, and overall confirmation purposes. Informants were recruited using flyers and notices. semi-structured interview guide used to elicit an understanding of the values, beliefs, and experiences of caregivers	• Sleeping with infants and children was viewed as a cultural caring behaviour promoting comfort, closeness and protection for infants, children, parents and caregivers<br>• The role of the nurse can impact accurate outcomes about SIDS and safe sleep practices.<br>• 56% of infants shared a sleep-surface with their caregiver<br>• younger women (20–22 years) with infants and children seem more likely to follow the teaching advice from the hospital regarding SIDS and safe sleep practices even if others in the family disagree<br>• grandmothers, aunts and older women in the family and local neighbourhood have much influence in how infants are put to sleep<br>• Informants stated that they did not nor have had health care professionals come to the community to provide services and promote heath through health education<br>• Informants reported that they would appreciate working with nurses and nursing students on a variety of health care issues in the community<br>• A common thread in all the data was that nursing intervention would be very welcomed regarding SIDS and safe sleep practice

References

1. Abdulrazzaq, Y.M.; Kendi, A.A.; Nagelkerke, N. Child care practice in the United Arab Emirates: the ESACCIPS study. *Acta Paediatrica* **2008**, *97*, 590-595, doi:doi:10.1111/j.1651-2227.2008.00758.x.
2. Aitken, M.E.; Rose, A.; Mullins, S.H.; Miller, B.K.; Nick, T.; Rettiganti, M.; Nabaweesi, R.; Whiteside-Mansell, L. Grandmothers' Beliefs and Practices in Infant Safe Sleep. *Matern. Child Health J.* **2016**, *20*, 1464-1471, doi:10.1007/s10995-016-1945-9.
3. Ajao, T.I.; Oden, R.P.; Joyner, B.L.; Moon, R.Y. Decisions of black parents about infant bedding and sleep surfaces: a qualitative study. *Pediatrics* **2011**, *128*, 494-502, doi:10.1542/peds.2011-0072.
4. Alahmadi, T.S.; Sobaihi, M.; Banjari, M.A.; Bakheet, K.M.A.; Modan Alghamdi, S.A.; Alharbi, A.S. Are Safe Sleep Practice Recommendations For Infants Being Applied Among Caregivers? *Cureus* **2020**, *12*, e12133, doi:10.7759/cureus.12133.
5. Alzahrani, R.M.; Algethami, N.E.; Alharbi, A.; Alharbi, N.; Abbas, R.; Alqthami, L.M.; Alshardi, I.; Aljaid, M. Awareness of Sudden Infant Death Syndrome Among Saudi Arabian Women in 2019: A Cross-Sectional Study. *Cureus* **2020**, *12*, e9768, doi:10.7759/cureus.9768.
6. Anderson, E.S.; Jackson, A.; Wailoo, M.P.; Petersen, S.A. Child care decisions: Parental choice or chance? *Child Care Health Dev.* **2002**, *28*, 391-401, doi:10.1046/j.1365-2214.2002.00288.x.
7. Anuntaseree, W.; Mo-Suwan, L.; Vasiknanonte, P.; Kuasirikul, S.; Ma-a-Lee, A.; Choprapawon, C. Factors associated with bed sharing and sleep position in Thai neonates. *Child Care Health Dev.* **2008**, *34*, 482-490, doi:10.1111/j.1365-2214.2008.00832.x.
8. Ateah, C.A.; Hamelin, K.J. Maternal bedsharing practices, experiences, and awareness of risks. *JOGNN J. Obstet. Gynecol. Neonatal Nurs.* **2008**, *37*, 274-281, doi:10.1111/j.1552-6909.2008.00242.x.
9. Austin, J.E.; Nashban, C.J.; Doering, J.J.; Davies, W.H. Prevention Messages in Parent-Infant Bed-Sharing: Message Source, Credibility, and Effectiveness. *Global pediatric health* **2017**, *4*, 2333794x17743403, doi:10.1177/2333794x17743403.
10. Baeis, M.G.; Miladinia, M.; Talayian, M.; Vahedi, M.; Vafayi, S.; Tashvighi, M. Prevalence and the relationship between characteristics and parental conditions with risk factors for Sudden Infant Death Syndrome (SIDS). *International Journal of Pediatrics* **2015**, *3*, 1135-1143, doi:10.22038/ijp.2015.5933.
11. Bailey, C. Breastfeeding mothers' experiences of bedsharing: A qualitative study. *Breastfeeding Review* **2016**, *24*, 33-40.
12. Bailey, C.; Tawia, S.; McGuire, E. Breastfeeding Duration and Infant Sleep Location in a Cohort of Volunteer Breastfeeding Counselors. *Journal of Human Lactation* **2020**, *36*, 354-364, doi:10.1177/0890334419851801.
13. Ball, H.L.; Moya, E.; Fairley, L.; et al. Infant care practices related to sudden infant death syndrome in South Asian and White British families in the UK. *Paediatric and Perinatal Epidemiology* **2012**, *26*, 3-12.
14. Ball, H.L.; Moya, E.; Fairley, L.; Westman, J.; Oddie, S.; Wright, J. Bed- and sofa-sharing practices in a UK biethnic population. *Pediatrics* **2012**, *129*, e673-e681, doi:10.1542/peds.2011-1964.
15. Barbir, I.; Ball, H.L.; Zakarija-Grković, I. Parental knowledge of safe infant sleep and sudden infant death syndrome is inadequate in Croatia. *Acta Paediatrica, International Journal of Paediatrics* **2020**, *109*, 1887-1888, doi:10.1111/apa.15207.
16. Beck, L.F.; Morrow, B.; Lipscomb, L.E.; et al. Prevalence of selected maternal behaviors and experiences, pregnancy risk assessment monitoring system (PRAMS), 1999. *Morbidity and Mortality Weekly Report (MMWR): CDC Surveillance Summaries* **2002**, *51*, 1-29.
17. Bombard, J.M.; Kortsmit, K.; Warner, L.; Shapiro-Mendoza, C.K.; Cox, S.; Kroelinger, C.D.; Parks, S.E.; Dee, D.L.; D'Angelo, D.V.; Smith, R.A.; et al. Vital Signs: Trends and Disparities in Infant Safe Sleep Practices - United States, 2009-2015. *MMWR: Morbidity & Mortality Weekly Report* **2018**, *67*, 39-46, doi:10.15585/mmwr.mm6701e1.
18. Brenner, R.A.; Simons-Morton, B.G.; Bhaskar, B.; et al. Infant-parent bed sharing in an inner-city population. *Archives of Pediatrics and Adolescent Medicine* **2003**, *157*, 33-39.
19. Broussard, D.; Sappenfield, W.; Goodman, D. The Black and White of Infant Back Sleeping and Infant Bed Sharing in Florida, 2004-2005. *Maternal & Child Health Journal* **2012**, *16*, 713-724, doi:10.1007/s10995-011-0768-y.
20. Caraballo, M.; Shimasaki, S.; Johnston, K.; Tung, G.; Albright, K.; Halbower, A.C. Knowledge, Attitudes, and Risk for Sudden Unexpected Infant Death in Children of Adolescent Mothers: A Qualitative Study. *Journal of Pediatrics* **2016**, *174*, 78-83.e72, doi:10.1016/j.jpeds.2016.03.031.
21. Cesar, J.A.; Acevedo, J.D.; Kaczan, C.R.; Venzo, J.C.P.; Costa, L.R.; da Silva, L.C.M.; Neumann, N.A. Identifying mothers' intention to place infant in supine sleep position: A population-based study. *Ciencia e Saude Coletiva* **2018**, *23*, 501-508, doi:10.1590/1413-81232018232.20732015.
22. Cesar, J.A.; Marmitt, L.P.; Carpena, M.X.; Pereira, F.G.; Neto, J.D.M.; Neumann, N.A.; Acevedo, J.D. Maternal Knowledge and Unsafe Baby Sleep Position: A Cross-Sectional Survey in Southern Brazil. *Matern. Child Health J.* **2019**, *23*, 183-190, doi:10.1007/s10995-018-2613-z.
23. Chung, E.K.; Hung, Y.Y.; Marchi, K.; Chavez, G.F.; Braveman, P. Infant sleep position: Associated maternal and infant factors. *Ambul. Pediatr.* **2003**, *3*, 234-239, doi:10.1367/1539-4409(2003)003<0234:ISPAMA>2.0.CO;2.
24. Chung-Park, C.M.S. Knowledge, opinions, and practices of infant sleep position among parents. *Mil. Med.* **2012**, *177*, 235-239.
25. Cole, R.; Young, J.; Kearney, L.; Thompson, J.M.D. Infant care practices and parent uptake of safe sleep messages: a cross-sectional survey in Queensland, Australia. *BMC pediatrics* **2020**, *20*, 1-13.

26. Cole, R.; Young, J.; Kearney, L.; Thompson, J.M.D. Reducing sleep-related infant mortality through understanding factors associated with breastfeeding duration: A cross-sectional survey. *Breastfeeding Review* **2020**, *28*.
27. Cole, R.; Young, J.; Kearney, L.; Thompson, J.M.D. Awareness of infant safe sleep messages and associated care practices: findings from an Australian cohort of families with young infants. *BMJ paediatrics open* **2021**, *5*.
28. Cole, R.; Young, J.; Kearney, L.; Thompson, J.M.D. Challenges parents encounter when implementing infant safe sleep advice. *Acta Paediatrica* **2021**, *110*, 3083-3093.
29. Colson, E.R.; Stille, C.J.; Payton, J.; Bernstein, B.; Dworkin, P. Not yet 'Back to Sleep': sleep position for infants in two inner-city clinics. *Ambulatory Child Health* **2000**, *6*, 269-275.
30. Colson, E.R.; Bergman, D.M.; Shapiro, E.; et al. Position for newborn sleep: associations with parents' perceptions of their nursery experience. *Birth* **2001**, *28*, 249-253.
31. Colson, E.R.; Levenson, S.; Rybin, D.; Calianos, C.; Margolis, A.; Colton, T.; Lister, G.; Corwin, M.J. Barriers to following the supine sleep recommendation among mothers at four centers for the Women, Infants, and Children Program. *Pediatrics* **2006**, *118*, e243-e250, doi:10.1542/peds.2005-2517.
32. Colson, E.R.; Rybin, D.; Smith, L.A.; et al. Trends and factors associated with infant sleeping position: the national infant sleep position study, 1993-2007. *Archives of Pediatrics and Adolescent Medicine* **2009**, *163*, 1122-1128.
33. Colson, E.R.; Willinger, M.; Rybin, D.; Heeren, T.; Smith, L.A.; Lister, G.; Corwin, M.J. Trends and factors associated with infant bed sharing, 1993-2010: The national infant sleep position study. *JAMA Pediatr.* **2013**, *167*, 1032-1037, doi:10.1001/jamapediatrics.2013.2560.
34. Colson, E.R.; Geller, N.L.; Heeren, T.; Corwin, M.J. Factors Associated With Choice of Infant Sleep Position. *Pediatrics* **2017**, *140*, doi:10.1542/peds.2017-0596.
35. Cornwell, B.; Yan, X.; Carlin, R.F.; Fu, L.; Wang, J.; Moon, R.Y. Social network influences on new mothers' infant sleep adjustments. *Social Science and Medicine* **2021**, *269*, doi:10.1016/j.socscimed.2020.113585.
36. Corwin, M.J.; Lesko, S.M.; Heeren, T.; et al. Secular changes in sleep position during infancy: 1995-1998. *Pediatrics* **2003**, *111*, 52-60.
37. Crane, D.; Ball, H.L. A qualitative study in parental perceptions and understanding of SIDS-reduction guidance in a UK bi-cultural urban community. *BMC Pediatrics* **2016**, *16*, 23, doi:10.1186/s12887-016-0560-7.
38. Cullen, A.; Kiberd, B.; McDonnell, M.; Mehanni, M.; Matthews, T.G.; O'Regan, M. Sudden infant death syndrome - Are parents getting the message? *Ir. J. Med. Sci.* **2000**, *169*, 40-43.
39. Cunningham, H.M.; Vally, H.; Bugeja, L. Bed-Sharing in the First 8 Weeks of Life: An Australian Study. *Matern. Child Health J.* **2018**, *22*, 556-564, doi:10.1007/s10995-017-2424-7.
40. da Silva, B.G.C.; da Silveira, M.F.; de Oliveira, P.D.; Domingues, M.R.; Neumann, N.A.; Barros, F.C.; Bertoldi, A.D. Prevalence and associated factors of supine sleep position in 3-month-old infants: findings from the 2015 Pelotas (Brazil) Birth Cohort. *BMC Pediatrics* **2019**, *19*, N.PAG-N.PAG, doi:10.1186/s12887-019-1534-3.
41. Damato, E.G.; Haas, M.C.; Czeck, P.; Dowling, D.A.; Barsman, S.G. Safe Sleep Infant Care Practices Reported by Mothers of Twins. *Adv Neonatal Care* **2016**, *16*, E3-14, doi:10.1097/anc.0000000000000332.
42. Douglas, T.A.; Buettner, P.G.; Whitehall, J. Maternal awareness of sudden infant death syndrome in North Queensland, Australia: An analysis of infant care practices. *Journal of Paediatrics and Child Health* **2001**, *37*, 441-445, doi:10.1046/j.1440-1754.2001.00670.x.
43. Duzinski, S.V.; Yuma-Guerrero, P.J.; Fung, A.; Brown, J.M.; Wheeler, T.; Barczyk, A.N.; Lawson, K.A. Sleep behaviors of infants and young children: associated demographic and acculturation characteristics among Hispanic teen mothers. *J Trauma Nurs* **2013**, *20*, 189-198, doi:10.1097/JTN.0000000000000011.
44. Efe, E.; Ak, G. Turkish mothers' who have preterm ınfants knowledge about risk factors of sudden ınfant death sendrome. *HealthMED* **2012**, *6*, 1137-1146.
45. Eisenberg, S.R.; Bair-Merritt, M.H.; Colson, E.R.; Heeren, T.C.; Geller, N.L.; Corwin, M.J. Maternal Report of Advice Received for Infant Care. *Pediatrics* **2015**, *136*, E315-E322, doi:10.1542/peds.2015-0551.
46. Epstein, J.; Jolly, C. Credibility gap? Parents' beliefs about reducing the risk of cot death. *Community Pract* **2009**, *82*, 21-24.
47. Erdoğan, Ç.; Turan, T. Risky Behaviors of Mothers with Infants on Sudden Infant Death Syndrome in Turkey. *J Pediatr Nurs* **2018**, *38*, e2-e6, doi:10.1016/j.pedn.2017.11.017.
48. Erick-Peleti, S.; Paterson, J.; Williams, M. Pacific Islands Families Study: maternal factors associated with cigarette smoking amongst a cohort of Pacific mothers with infants. *N. Z. Med. J.* **2007**, *120*.
49. Fernandes, S.C.; de Luca, F.; Fonseca, S.; Oliveira, F.; Areias, M. Sudden Infant Death Syndrome: What Healthcare Professionals and Parents Know About How to Prevent it in Portugal. *Yale J Biol Med* **2020**, *93*, 475-485.
50. Ford, R.P.K.; Schluter, P.J.; Cowan, S. Changes to infant sleep practices in Canterbury. *New Zealand Medical Journal* **2000**, *113*, 8-10.
51. Fowler, A.J.; Evans, P.W.; Etchegaray, J.M.; et al. Safe sleep practices and sudden infant death syndrome risk reduction: NICU and well-baby nursery graduates. *Clinical Pediatrics* **2013**, *52*, 1044-1053.
52. Galland, B.C.; Gray, A.; Sayers, R.M.; Heath, A.-L.M.; Lawrence, J.; Taylor, R.; Taylor, B.J. Safe sleep practices in a New Zealand community and development of a Sudden Unexpected Death in Infancy (SUDI) risk assessment instrument. *BMC Pediatrics* **2014**, *14*, 263-263, doi:10.1186/1471-2431-14-263.
53. Gaydos, L.M.; Blake, S.C.; Gazmararian, J.A.; Woodruff, W.; Thompson, W.W.; Dalmida, S.G. Revisiting Safe Sleep Recommendations for African-American Infants: Why Current Counseling is Insufficient. *Maternal & Child Health Journal* **2015**, *19*, 496-503, doi:10.1007/s10995-014-1530-z.

54. Gibson, E.; Greenspan, J.S.; Rubin, S.; Dembofsky, C.A. Infant Sleep Position Practices 2 Years Into the "Back to Sleep" Campaign. *Clinical Pediatrics* **2000**, *39*, 285-289.
55. Goodstein, M.H.; Bell, T.; Krugman, S.D. Improving infant sleep safety through a comprehensive hospital-based program. *Clinical Pediatrics* **2015**, *54*, 212-221, doi:10.1177/0009922814566928.
56. Haas, M.C.; Dowling, D.; Damato, E.G. Adherence to Safe Sleep Recommendations by Families With Higher-order Multiples. *Adv Neonatal Care* **2017**, *17*, 407-416, doi:10.1097/anc.0000000000000416.
57. Hamadneh, S.; Kassab, M.; Hamadneh, J.; Amarin, Z. Sudden unexpected infant death in Jordan and the home environment. *Pediatr Int* **2016**, *58*, 1333-1336, doi:10.1111/ped.13016.
58. Hannan, K.E.; Smith, R.A.; Barfield, W.D.; Hwang, S.S. Association between Neonatal Intensive Care Unit Admission and Supine Sleep Positioning, Breastfeeding, and Postnatal Smoking among Mothers of Late Preterm Infants. *Journal of Pediatrics* **2020**, *227*, 114-120.e111, doi:10.1016/j.jpeds.2020.07.053.
59. Hauck, F.R.; Signore, C.; Fein, S.B.; Raju, T.N.K. Infant sleeping arrangements and practices during the first year of life. *Pediatrics* **2008**, *122*, S113-S120, doi:10.1542/peds.2008-1315o.
60. Hauck, F.R.; Tanabe, K.O.; McMurry, T.; Moon, R.Y. Evaluation of bedtime basics for babies: a national crib distribution program to reduce the risk of sleep-related sudden infant deaths. *J Community Health* **2015**, *40*, 457-463, doi:10.1007/s10900-014-9957-0.
61. Hirabayashi, M.; Yoshinaga, M.; Nomura, Y.; Ushinohama, H.; Sato, S.; Tauchi, N.; Horigome, H.; Takahashi, H.; Sumitomo, N.; Shiraishi, H.; et al. Environmental risk factors for sudden infant death syndrome in Japan. *Eur J Pediatr* **2016**, *175*, 1921-1926, doi:10.1007/s00431-016-2786-7.
62. Homer, C.; Armari, E.; Fowler, C. Bed-sharing with infants in a time of SIDS awareness. *Neonatal, Paediatric & Child Health Nursing* **2012**, *15*, 3-7.
63. Hussain, S.; Lowell, G.S.; Roehler, D.R.; Quinlan, K.P.; Tandon, S.D.; Schwartz, L. You can have your breastmilk and safe sleep too: a preliminary analysis of infant safe sleep data in a Midwestern home visiting program. *Inj Epidemiol* **2018**, *5*, 14, doi:10.1186/s40621-018-0138-y.
64. Hutchison, L.; Stewart, A.W.; Mitchell, E.A. SIDS-protective infant care practices among Auckland, New Zealand mothers. *New Zealand Med. J.* **2006**, *119*.
65. Hutchison, L.; Stewart, A.; Mitchell, E. Infant sleep position, head shape concerns, and sleep positioning devices. *Journal of paediatrics and child health* **2007**, *43*, 243-248, doi:10.1111/j.1440-1754.2007.01054.x.
66. Hutchison, B.L.; Stewart, A.W.; Mitchell, E.A. The prevalence of cobedding and SIDS-related child care practices in twins. *Eur J Pediatr* **2010**, *169*, 1477-1485.
67. Hutchison, L.; Thompson, J.M.D.; Mitchell, E.A. Infant care practices related to sudden unexpected death in infancy: a 2013 survey. *New Zealand Medical Journal* **2015**, *128*, 15-22.
68. Hwang, S.S.; Barfield, W.D.; Smith, R.A.; Morrow, B.; Shapiro-Mendoza, C.K.; Prince, C.B.; Smith, V.C.; McCormick, M.C. Discharge timing, outpatient follow-up, and home care of late-preterm and early-term infants. *Pediatrics* **2013**, *132*, 101-108, doi:10.1542/peds.2012-3892.
69. Hwang, S.S.; Smith, R.A.; Barfield, W.D.; Smith, V.C.; McCormick, M.C.; Williams, M.A. Supine sleep positioning in preterm and term infants after hospital discharge from 2000 to 2011. *Journal of Perinatology* **2016**, *36*, 787-793, doi:10.1038/jp.2016.80.
70. Inbar, Z.; Meibar, R.; Shehada, S.; Irena, V.; Rubin, S.; Rishpon, S. 'Back to sleep': parents compliance with the recommendation on the most appropriate sleeping position of infants, Haifa District, Israel, 2001. *Preventive Medicine* **2005**, *40*, 765-768.
71. Joyner, B.L.; Oden, R.P.; Moon, R.Y. Reasons for Pacifier Use and Non-Use in African-Americans: Does Knowledge of Reduced SIDS Risk Change Parents' Minds? *J Immigr Minor Health* **2016**, *18*, 402-410, doi:10.1007/s10903-015-0206-0.
72. Kelmanson, I. Swaddling: Maternal Option and Sleep Behaviour in Two-month-old Infants. *Child Care in Practice* **2013**, *19*, 36-48, doi:10.1080/13575279.2012.712035.
73. Kihlström, L.; Agu, N.; Dorjulus, B.; Prieto, C.; Chandran, V.; Alastre, S.; Rojas, D.; Birriel, P.C.; Marshall, J. Using Photovoice to Understand Perspectives on Safe Infant Sleep Practices among Caregivers Enrolled in Perinatal Home Visiting. *Journal of Social Service Research* **2020**, *47*, 357-368, doi:10.1080/01488376.2020.1816594.
74. Konstat-Korzenny, E.; Cohen-Welch, A.; Fonseca-Portilla, R.; Morgenstern-Kaplan, D. Sudden Unexpected Infant Death: Review and Analysis of Adherence to Recommendations. *Cureus* **2019**, *11*, e6076, doi:10.7759/cureus.6076.
75. Krouse, A.; Craig, J.; Watson, U.; Matthews, Z.; Kolski, G.; Isola, K. Bed-sharing influences, attitudes, and practices: Implications for promoting safe infant sleep. *Journal of Child Health Care* **2012**, *16*, 274-283, doi:10.1177/1367493511432300.
76. Kuhlmann, Z.; Kuhlmann, S.; Schunn, C.; Klug, B.F.; Greaves, T.; Foster, M.; Ahlers-Schmidt, C.R. Collaborating with obstetrical providers to promote infant safe sleep guidelines. *Sleep health* **2016**, *2*, 219-224, doi:10.1016/j.sleh.2016.06.003.
77. Lahr, M.B.; Rosenberg, K.D.; Lapidus, J.A. Health departments do it better: Prenatal care site and prone infant sleep position. *Matern. Child Health J.* **2005**, *9*, 165-172, doi:10.1007/s10995-005-4868-4.
78. Lahr, M.B.; Rosenberg, K.D.; Lapidus, J.A. Bedsharing and maternal smoking in a population-based survey of new mothers. *Pediatrics* **2005**, *116*, e530-542.
79. Lahr, M.B.; Rosenberg, K.D.; Lapidus, J.A. Maternal-infant bedsharing: Risk factors for bedsharing in a population-based survey of new mothers and implications for SIDS risk reduction. *Matern. Child Health J.* **2007**, *11*, 277-286, doi:10.1007/s10995-006-0166-z.

80. Lau, A.; Hall, W. Safe sleep, day and night: mothers' experiences regarding infant sleep safety. *Journal of Clinical Nursing* **2016**, *25*, 2816-2826, doi:10.1111/jocn.13322.
81. Martiniuk, A.; Jacob, J.; Faruqui, N.; Yu, W. Positional plagiocephaly reduces parental adherence to SIDS Guidelines and inundates the health system. *Child Care Health Dev.* **2016**, *42*, 941-950, doi:10.1111/cch.12386.
82. Mathews, A.; Joyner, B.; Oden, R.; Alamo, I.; Moon, R. Comparison of Infant Sleep Practices in African-American and US Hispanic Families: Implications for Sleep-Related Infant Death. *Journal of Immigrant & Minority Health* **2015**, *17*, 834-842, doi:10.1007/s10903-014-0016-9.
83. Miladinia, M.; Baraz, S.; Nouri, E.M. Sudden infant death syndrome: Risk factors and the relationship between them. *International Journal of Pediatrics* **2015**, *3*, 1103-1110, doi:10.22038/ijp.2015.5724.
84. Möllborg, P.; Wennergren, G.; Norvenius, S.G.; Alm, B. Bed-sharing among six-month-old infants in western Sweden. *Acta Paediatrica, International Journal of Paediatrics* **2011**, *100*, 226-230, doi:10.1111/j.1651-2227.2010.02008.x.
85. Moon, R.Y.; Oden, R.P.; Joyner, B.L.; et al. Qualitative analysis of beliefs and perceptions about sudden infant death syndrome in African-American mothers: implications for safe sleep recommendations. *Journal of Pediatrics* **2010**, *157*, 92-97.
86. Moon, R.Y.; Carlin, R.F.; Cornwell, B.; Mathews, A.; Oden, R.P.; Cheng, Y.I.; Fu, L.Y.; Wang, J. Implications of Mothers' Social Networks for Risky Infant Sleep Practices. *Journal of Pediatrics* **2019**, *212*, 151-158.e152, doi:10.1016/j.jpeds.2019.05.027.
87. Moon, R.Y.; Omron, R. Determinants of infant sleep position in an urban population. *Clinical Pediatrics* **2002**, *41*, 569-573.
88. Nelson, E.A.; Taylor, B.J.; ICCPS Study Group. International Child Care Practices Study: infant sleep position and parental smoking. *Early Human Development* **2001**, *64*, 7-20.
89. Nelson, E.A.S.; Taylor, B.J.; Jenik, A.; Vance, J.; Walmsley, K.; Pollard, K.; Freemantle, M.; Ewing, D.; Einspieler, C.; Engele, H.; et al. International Child Care Practices Study: infant sleeping environment. *Early Human Development* **2001**, *62*, 43-55, doi:10.1016/S0378-3782(01)00116-5.
90. Nelson, E.A.S.; Yu, L.; Williams, S. International Child Care Practices Study: breastfeeding and pacifier use. *Journal of Human Lactation* **2005**, *21*, 289-295.
91. Norton, P.J.; Grellner, K.W. A Retrospective Study on Infant Bed-Sharing in a Clinical Practice Population. *Maternal & Child Health Journal* **2011**, *15*, 507-513, doi:10.1007/s10995-010-0600-0.
92. Nongkynrih, R.; Cutinho, S.P.; D'Almeida, V. A Study on Knowledge and Practice on Maternal Bed sharing Among Mothers in A Selected Hospital at Mangalore. *Nitte University Journal of Health Science* **2017**, *7*, 3-7.
93. Oden, R.P.; Powell, C.; Sims, A.; et al. Swaddling: will it get babies onto their backs for sleep? *Clinical Pediatrics* **2012**, *51*, 254-259.
94. Osberg, S.; Kalstad, T.G.; Stray-Pedersen, A. Norwegian parents avoid placing infants in prone sleeping positions but frequently share beds in hazardous ways. *Acta Paediatrica* **2021**, doi:10.1111/apa.15797.
95. Panaretto, K.S.; Smallwood, V.E.; Cole, P.; Elston, J.; Whitehall, J.S. Sudden infant death syndrome risk factors in north Queensland: A survey of infant-care practices in Indigenous and non-Indigenous women. *Journal of Paediatrics and Child Health* **2002**, *38*, 129-134, doi:10.1046/j.1440-1754.2002.00759.x.
96. Paterson, J.; Tukuitonga, C.; Butler, S.; Williams, M. Infant bed-sharing among Pacific families in New Zealand. *New Zealand Medical Journal* **2002**, *115*, 241-243.
97. Pease, A.; Ingram, J.; Blair, P.S.; Fleming, P.J. Factors influencing maternal decision-making for the infant sleep environment in families at higher risk of SIDS: A qualitative study. *BMJ Paediatrics Open* **2017**, *1*, doi:10.1136/bmjpo-2017-000133.
98. Pease, A.S.; Blair, P.S.; Ingram, J.; Fleming, P.J. Mothers' knowledge and attitudes to sudden infant death syndrome risk reduction messages: results from a UK survey. *Arch Dis Child* **2018**, *103*, 33-38, doi:10.1136/archdischild-2017-312927.
99. Phares, T.M.; Morrow, B.; Lansky, A.; et al. Surveillance for disparities in maternal health-related behaviors - selected states, Pregnancy Risk Assessment Monitoring System (PRAMS), 2000-2001. *Morbidity and Mortality Weekly Report (MMWR)* **2004**, *53*, 1-13.
100. Pretorius, K.; Choi, E.; Kang, S.; Mackert, M. Sudden Infant Death Syndrome on Facebook: Qualitative Descriptive Content Analysis to Guide Prevention Efforts. *Journal of Medical Internet Research* **2020**, *22*, N.PAG-N.PAG, doi:10.2196/18474.
101. Provini, L.E.; Corwin, M.J.; Geller, N.L.; Heeren, T.C.; Moon, R.Y.; Rybin, D.V.; Shapiro-Mendoza, C.K.; Colson, E.R. Differences in Infant Care Practices and Smoking among Hispanic Mothers Living in the United States. *Journal of Pediatrics* **2017**, *182*, 321-326.e321, doi:10.1016/j.jpeds.2016.11.053.
102. Raines, D.A. Factors That Influence Parents' Adherence to Safe Sleep Guidelines. *JOGNN - Journal of Obstetric, Gynecologic, and Neonatal Nursing* **2018**, *47*, 316-323, doi:10.1016/j.jogn.2018.01.010.
103. Roberts, H.; Upton, D. New mothers' knowledge of sudden infant death syndrome. *British Journal of Midwifery* **2000**, *8*, 147-150.
104. Robida, D.; Moon, R.Y. Factors influencing infant sleep position: decisions do not differ by SES in African-American families. *Arch Dis Child* **2012**, *97*, 900-905, doi:10.1136/archdischild-2011-301360.
105. Rohana, J.; Ishak, S.; Wan Nurulhuda, W.M.Z. Sudden infant death syndrome: Knowledge and practise in parents of preterm infants. *Pediatr Int* **2018**, *60*, 710-713, doi:10.1111/ped.13605.

106. Ruiz Botia, I.; Cassanello Peñarroya, P.; Díez Izquierdo, A.; Martínez Sánchez, J.M.; Balaguer Santamaria, A. Sudden infant death syndrome: Do the parents follow the recommendations? *Anales de Pediatria* **2020**, *92*, 222-228, doi:10.1016/j.anpedi.2019.06.011.
107. Salm Ward, T.; Ngui, E. Factors Associated with Bed-Sharing for African American and White Mothers in Wisconsin. *Matern. Child Health J.* **2015**, *19*, 720-732, doi:10.1007/s10995-014-1545-5.
108. Salm Ward, T.C.; Kanu, F.A.; Anderson, A.K. Trends and Factors Associated with Breastfeeding and Infant Sleep Practices in Georgia. *J Community Health* **2018**, *43*, 496-507, doi:10.1007/s10900-017-0442-4.
109. Salm Ward, T.C.; Robb, S.W.; Kanu, F.A. Prevalence and Characteristics of Bed-Sharing Among Black and White Infants in Georgia. *Matern. Child Health J.* **2016**, *20*, 347-362, doi:10.1007/s10995-015-1834-7.
110. Sawaguchi, T.; Nishida, H.; Fukui, F.; Horiuchi, T.; Nelson, E.A.S. Study on social responses (encouraging public awareness) to sudden infant death syndrome: Evaluation of SIDS prevention campaigns. *Forensic Sci. Int.* **2002**, *130*, S78-S80.
111. Schluter, P.J.; Paterson, J.; Percival, T. Infant care practices associated with sudden infant death syndrome: findings from the Pacific Islands Families study. *Journal of Paediatrics and Child Health* **2007**, *43*, 388-393.
112. Schluter, P.J.; Young, J. Reducing the risk of SIDS: what infant care practices are being used by primary caregivers in Queensland? *Neonatal, Paediatric and Child Health Nursing* **2002**, *5*, 26-33.
113. Shapiro-Mendoza, C.K.; Colson, E.R.; Willinger, M.; et al. Trends in infant bedding use: National Infant Sleep Position Study, 1993-2010. *Pediatrics* **2015**, *135*, 10-17.
114. Shields, L.B.E.; Hunsaker, D.M.; Muldoon, S.; Corey, T.S.; Spivack, B.S. Risk factors associated with sudden unexplained infant death: A prospective study of infant care practices in Kentucky. *Pediatrics* **2005**, *116*, e13-e20, doi:10.1542/peds.2004-2333.
115. Sivan, Y.; Reisner, S.; Amitai, Y.; Wasser, J.; Nehama, H.; Tauman, R. Effect of religious observance on infants' sleep position in the Jewish population. *Journal of Paediatrics and Child Health* **2004**, *40*, 534-539, doi:10.1111/j.1440-1754.2004.00458.x.
116. Smith, M.G.; Liu, J.H.; Helms, K.H.; Wilkerson, K.L. Racial differences in trends and predictors of infant sleep positioning in South Carolina, 1996-2007. *Matern. Child Health J.* **2012**, *16*, 72-82, doi:10.1007/s10995-010-0718-0.
117. Smith, L.A.; Geller, N.L.; Kellams, A.L.; Colson, E.R.; Rybin, D.V.; Heeren, T.; Corwin, M.J. Infant Sleep Location and Breastfeeding Practices in the United States, 2011-2014. *Acad. Pediatr.* **2016**, doi:10.1016/j.acap.2016.01.021.
118. Smylie, J.; Fell, D.B.; Chalmers, B.; Sauve, R.; Royle, C.; Allan, B.; O'Campo, P.; Maternity Experiences Study, G. Socioeconomic Position and Factors Associated With Use of a Nonsupine Infant Sleep Position: Findings From the Canadian Maternity Experiences Survey. *American Journal of Public Health* **2014**, *104*, 539-547, doi:10.2105/ajph.2012.301061.
119. Specker, B.L.; Minett, M.; Beare, T.; Poppinga, N.; Carpenter, M.; Munger, J.; Strasser, K.; Ahrendt, L. Safe Sleep Behaviors Among South Dakota Mothers and the Role of the Healthcare Provider. *S D Med* **2020**, *73*, 152-162.
120. Sperhake, J.P.; Zimmermann, I.; Püschel, K. Current recommendations on infants' sleeping position are being followed-initial results of a population-based sentinel study on risk factors for SIDS, 1996-2006, in Hamburg, Germany. *Int. J. Leg. Med.* **2009**, *123*, 41-45, doi:10.1007/s00414-008-0298-3.
121. Strömberg Celind, F.; Wennergren, G.; Möllborg, P.; Goksör, E.; Alm, B. Area-based study shows most parents follow advice to reduce risk of sudden infant death syndrome. *Acta Paediatrica* **2017**, *106*, 579-585, doi:10.1111/apa.13711.
122. Tipene-Leach, D.; Hutchison, L.; Tangiora, A.; Rea, C.; White, R.; Stewart, A.; Mitchell, E. SIDS-related knowledge and infant care practices among Māori mothers. *New Zealand Medical Journal* **2010**, *123*, 88-96.
123. Tirosh, E.; Becker, T.; Mansour, Y.; Cohen, A.; Jaffe, M. Sleep position, bedding and heating practices in high- and low-risk ethnic groups for unexpected death in infancy (UDI). *Eur. J. Epidemiol.* **2000**, *16*, 281-286, doi:10.1023/A:1007682605820.
124. Tully, K.; Holditch-Davis, D.; Brandon, D. The Relationship Between Planned and Reported Home Infant Sleep Locations Among Mothers of Late Preterm and Term Infants. *Maternal & Child Health Journal* **2015**, *19*, 1616-1623, doi:10.1007/s10995-015-1672-7.
125. van Sleuwen, B.E.; L'Hoir, M.P.; Engelberts, A.C.; Westers, P.; Schulpen, T.W.J. Infant care practices related to cot death in Turkish and Moroccan families in the Netherlands. *Arch Dis Child* **2003**, *88*, 784-788.
126. Vernacchio, L.; Corwin, M.J.; Lesko, S.M.; Vezina, R.M.; Hunt, C.E.; Hoffman, H.J.; Willinger, M.; Mitchell, A.A. Sleep position of low birth weight infants. *Pediatrics* **2003**, *111*, 633-640, doi:10.1542/peds.111.3.633.
127. Varghese, S.; Gasalberti, D.; Ahern, K.; Chang, J.C. An analysis of attitude toward infant sleep safety and SIDS risk reduction behavior among caregivers of newborns and infants. *Journal of Perinatology* **2015**, *35*, 970-973, doi:10.1038/jp.2015.111.
128. Vilvens, H.L.; Vaughn, L.M.; Southworth, H.; Denny, S.A.; Gittelman, M.A. Personalising Safe Sleep Messaging for Infant Caregivers in the United States. *Health and Social Care in the Community* **2020**, *28*, 891-902, doi:10.1111/hsc.12920.
129. Von Kohorn, I.; Corwin, M.J.; Rybin, D.V.; Heeren, T.C.; Lister, G.; Colson, E.R. Influence of prior advice and beliefs of mothers on infant sleep position. *Archives of Pediatrics and Adolescent Medicine* **2010**, *164*, 363-369.
130. Walcott, R.L.; Salm Ward, T.C.; Ingels, J.B.; Llewellyn, N.A.; Miller, T.J.; Corso, P.S. A Statewide Hospital-Based Safe Infant Sleep Initiative: Measurement of Parental Knowledge and Behavior. *J Community Health* **2018**, *43*, 534-542, doi:10.1007/s10900-017-0449-x.

131. Wennergren, G.; Strömberg Celind, F.; Goksör, E.; Alm, B. Swedish survey of infant sleep practices showed increased bed-sharing and positive associations with breastfeeding. *Acta paediatrica (Oslo, Norway : 1992)* **2021**, *110*, 1835-1841, doi:10.1111/apa.15719.
132. Willinger, M.; Ko, C.W.; Hoffman, H.J.; Kessler, R.C.; Corwin, M.J. Factors associated with caregivers' choice of infant sleep position, 1994-1998: The national infant sleep position study. *J. Am. Med. Assoc.* **2000**, *283*, 2135-2142.
133. Wilson, C.E. Cree infant care practices and sudden infant death syndrome. *Canadian Journal of Public Health* **2000**, *91*, 133-136.
134. Woods, N.K.; Ahlers-Schmidt, C.R.; Wipperman, J.; Williams, T. Comparing Self-Reported Infant Safe Sleep From Community- and Health Care-Based Settings. *Journal of Primary Care and Community Health* **2015**, *6*, 205-210, doi:10.1177/2150131914567967.
135. Wright, C.; Beard, H.; Cox, J.; et al. Parents' choice of non-supine sleep position for newborns: a cross-sectional study. *British Journal of Midwifery* **2014**, *22*, 625-629.
136. Yikilkan, H.; Ünalan, P.C.; Cakir, E.; Ersu, R.H.; Cifcili, S.; Akman, M.; Uzuner, A.; Dagli, E. Sudden infant death syndrome: How much mothers and health professionals know. *Pediatr Int* **2011**, *53*, 24-28, doi:10.1111/j.1442-200X.2010.03202.x.
137. Zoucha, R.; Walters, C.A.; Colbert, A.M.; Carlins, E.; Smith, E. Exploring Safe Sleep and SIDS Risk Perception in an African-American Community: Focused Ethnography. *Public Health Nurs* **2016**, *33*, 206-213, doi:10.1111/phn.12235.
